# Clinical Applications and Measurement Properties of the Digitized Archimedes Spiral Drawing Test: A Scoping Review

**DOI:** 10.1002/mdc3.70278

**Published:** 2025-08-07

**Authors:** Stella Wang, Tim Schwirtlich, David McLaughlin, Molly Beestrum, Allen W. Heinemann

**Affiliations:** ^1^ Institute for Public Health and Medicine (IPHAM), Center for Education in Health Sciences, Health Sciences Integrated Program Northwestern University Feinberg School of Medicine Chicago Illinois USA; ^2^ Cerebral Innovations St. Petersburg Florida USA; ^3^ Galter Health Science Library Galter Health Science Library Northwestern University Feinberg School of Medicine Chicago Illinois USA; ^4^ Physical Medicine and Rehabilitation, Northwestern University Feinberg School of Medicine and Shirley Ryan AbilityLab Chicago Illinois USA

**Keywords:** digitized spiral drawing test, fine motor function, clinical outcome measurement, measurement properties

## Abstract

**Background:**

The digitized Archimedes spiral drawing test (DAST) provides an objective approach for assessing tremor in Parkinson's disease (PD) and essential tremor (ET). Despite its growing adoption in patient care settings and clinical research, the measurement properties of the digitized spiral drawing test beyond tremor are underexplored. Additionally, methodological variability limits its clinical standardization and broader clinical application.

**Objective:**

This scoping review evaluates various applications of the digitized spiral drawing test as a clinical outcome measurement instrument in adult populations, emphasizing its reliability, validity, and responsiveness.

**Method:**

This scoping review examined literature published in MEDLINE, Embase, CINAHL, PsycINFO, Scopus, and Web of Science, including studies on adults (≥18 years) using the DAST, to assess fine motor function.

**Results:**

Of 120 included studies, 84 focused on PD or ET, with emerging applications in other conditions. Digital tablets were the most used administration method (66.6%), but task design, metrics, and analysis varied. Measurement properties were reported in fifty‐two studies (43.3%): 15 on reliability, 45 on validity, and 6 on responsiveness. Results demonstrated consistent reliability, strong correlations with gold‐standard clinical measures, and sensitivity to interventions.

**Conclusions:**

The digitized spiral drawing test shows strong promise as a reliable and valid tool for assessing fine motor function in clinical and research settings. However, significant variability in devices, drawing tasks, and analysis methods across studies highlights the need for standardized protocols. Future research should focus on harmonizing methods, expanding its application across different conditions, and ensuring its clinical relevance as a measurement instrument.

Assessing fine motor function is an important component of neurological health evaluation. It is critical for diagnosing, monitoring, and tracking the progression of disorders such as Parkinson's disease (PD) and essential tremor (ET). Among the available tools, the Archimedes spiral drawing test has been widely used for decades due to its simplicity and effectiveness.^1^, ^2^ This test usually involves the participant drawing a spiral. The spiral is subsequently evaluated by a trained clinician using a visual rating scale, which is typically scored on an ordinal scale (eg, 0–10 on the Bain and Findley spiral score) to assess drawing quality and determine tremor severity.^3^ Although visual rating is an effective method, it relies on subjective judgments, which are inherently prone to inter‐ and intra‐rater variability.^4^ For example, inter‐rater reliability (Cohen's weighted kappa κ) ranged from 0.56 to 0.90 and intra‐rater reliability from 0.36 to 0.88 in participants with ET.^3^ The reliability of visual rating is particularly low for mild tremors because subtle motor irregularities are challenging to distinguish from normal movement.^3^, ^4^, ^5^ These limitations highlight the need for objective and precise assessment methods.

With digital advancements, the paper‐based spiral drawing test has evolved into high‐resolution, sensor‐based platforms since the mid‐1990s.^6^, ^7^, ^8^ Modern graphic tablets and touchscreen devices can accurately capture position, time, pressure, tilt, and in‐air movements. For example, Wacom (Wacom Technology Corporation, Vancouver, WA) tablets sample at 133 to 200 Hz and provide resolutions up to 5080 LPI and 8192 pressure levels, which is comparable to high‐end imaging.^9^ These data can be analyzed using spatiotemporal transformations, spectral analysis, or machine learning (ML) to produce detailed movement information that helps detect motor irregularities, track disease progression, and evaluate treatments.^10^, ^11^, ^12^, ^13^, ^14^

Although far more sensitive and objective than clinician‐rated tests, the digitized Archimedes spiral drawing test (DAST) does not guarantee greater reliability or validity. Sensor‐based data cannot fully eliminate user bias and systematic errors. For example, a precise digital system might accurately track minor variations in drawing trajectory but could still produce unreliable results if these variations are measured inconsistently across devices or testing environments. Haubenberger and colleagues identified several limitations that may impact the measurement outcome, including variability in drawing instruction, differences in device recording frequency and accuracy, and challenges with data standardization.^15^

ML and artificial intelligence (AI) models can introduce additional complexity to DAST analysis. Although these models may improve diagnostic accuracy, their clinical value depends on data quality, analytical methods, and risks like bias or overfitting.^16^, ^17^, ^18^, ^19^ A pure algorithm‐driven approach may also lack real‐world relevance. Evaluating DAST's measurement properties is therefore essential to confirm its reliability and support its use as a standardized tool for assessing motor impairments.^20^

This scoping review aims to map peer‐reviewed literature on the DAST's clinical applications and measurement properties in adults, focusing on its use as a measurement tool rather than a diagnostic test. A preliminary search revealed no prior scoping or systematic reviews on this topic. Using the Consensus‐Based Standards for the Selection of Health Measurement Instruments (PRISMA‐COSMIN) framework,^21^, ^22^ this review categorizes evidence on validity, reliability, and responsiveness; identifies research gaps; and supports efforts to standardize the DAST.

## Patients and Methods

### Protocol and Registration

The protocol was developed following JBI methodology for scoping reviews and registered at Open Science Framework (OSF) on November 8, 2024 (https://doi.org/10.17605/OSF.IO/WEUTR).^23^ The findings are reported in accordance with the Preferred Reporting Items for Systematic Reviews and Meta‐Analyses extension for Scoping Reviews.^24^

### Eligibility Criteria

Studies were included if they assessed the clinical applications or measurement properties of digital spiral drawings. Eligible studies involved adults (≥18 years) and used quantitative metrics rather than visual ratings. The review covers the use of the DAST in both clinical and research settings. Only peer‐reviewed journal articles in English were included due to resource constraints, whereas gray literature was excluded to prioritize rigor and quality. As a scoping review, there is no set sample size, as the goal is to comprehensively map existing literature rather than perform a power analysis. All identified sources were included to provide a thorough overview of the current evidence on DAST's clinical applications and measurement properties.

### Information Sources

To identify potentially relevant documents, the following bibliographic databases were searched without date restrictions: MEDLINE (via PubMed), Embase (via Elsevier), CINAHL (via EBSCO), PsycINFO (via EBSCO), Scopus (via Elsevier), and Web of Science (via Clarivate Analytics). The initial search strategies were drafted by an experienced librarian and refined through collaborative discussions between authors to ensure comprehensive coverage.

### Search

The search strategy is designed to broadly capture published studies using the DAST. The search terms include both keywords and Medical Subject Heading (MeSH) terms, using Boolean logic, truncation, and wildcards to maximize the retrieval of relevant articles. No language or date limits were applied, with the goal of ensuring a comprehensive search. See Table S1 for the search terms used and the detailed search strategy.

### Selection of Sources of Evidence

The final search was conducted on November 1, 2024. Duplicate records were identified and removed, and the final search results were exported into management software (Rayyan, https://www.rayyan.ai/) for 2‐level screening.^25^ First, titles and abstracts were screened independently by 3 reviewers, with conflicts identified and resolved by a third reviewer. Second, full texts of all papers included at title and abstract screening stage were screened using the same processes. Excluded sources at full‐text screening and reasons for exclusion are recorded in an Excel spreadsheet (Table S2) and reported in the PRISMA flowchart.

### Data‐Charting Process and Data Items

A data‐charting form was initially developed by 1 reviewer and refined through pilot testing during the extraction process to ensure clarity, comprehensiveness, and alignment with the study objectives. The final version is provided in Table S3. Extracted data were continuously reviewed against the protocol criteria to identify ambiguities or gaps. Key data extraction items included study details (author, year of publication, country, setting, and sample size) and participant characteristics. Additionally, information on data acquisition and analysis methods, assessed measurement properties, key results, and clinical implications was recorded.

### Synthesis of Results

The studies were labeled by the populations they analyzed, and the type of settings, populations, and study designs for each group were summarized, along with the measures used and broad findings. A subset of studies that evaluated measurement properties was further examined and grouped based on the specific types of measurement properties analyzed (eg, reliability, validity, responsiveness). For each group, the populations studied, study designs, measures employed, and broad findings were summarized.

## Results

### Selection of Sources of Evidence

**FIG. 1:**
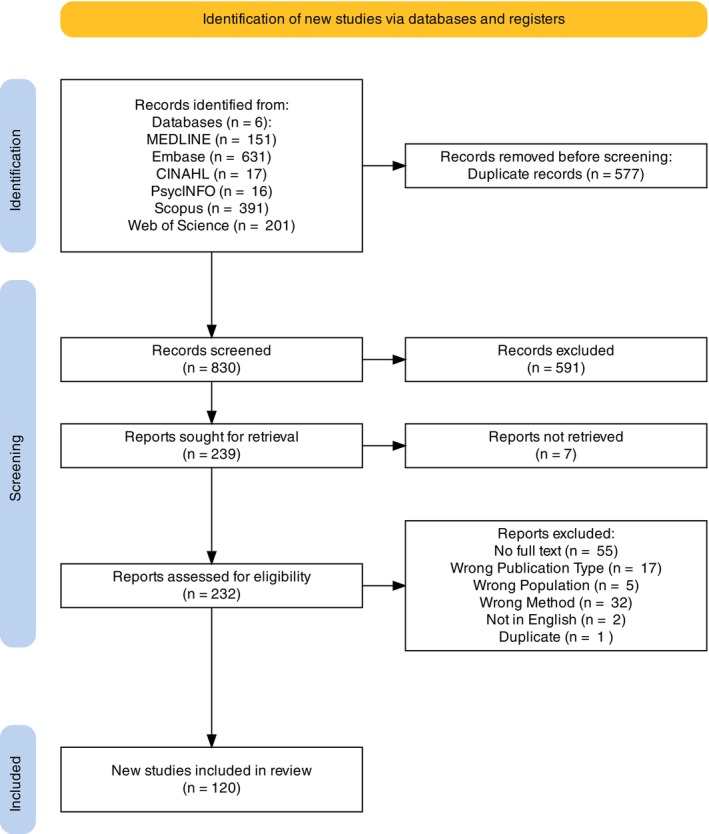
Flow diagram of article selection process based on Preferred Reporting Items for Systematic Reviews and Meta‐analyses Extension for Scoping Reviews (PRISMA‐ScR).

The 6 database searches yielded 1407 citations. After 577 duplicates were removed, 830 unique citations were screened. Titles and abstracts were reviewed, leading to the exclusion of 591 papers due to irrelevant content. The remaining citations underwent detailed full‐text assessment, and 119 were excluded, leaving 120 citations in this review. A PRISMA flowchart (Fig. 1) provides a detailed overview of the number of citations at each stage and the reasons for exclusion.

### Publication Trend

**FIG. 2:**
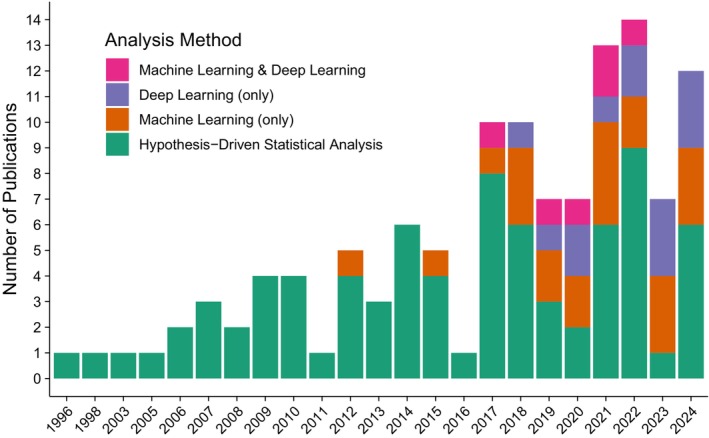
Publication trend in studies on the DAST (digitized Archimedes spiral drawing test) since the mid‐1990s.

The analysis of publication trends shows a steady increase in studies on the DAST since the mid‐1990s (Fig. 2). Until the mid‐2010s, most studies used hypothesis‐driven statistical analyses. This approach helped lay the groundwork for digitized methods in assessing fine motor function. The number of publications on the DAST has increased significantly since 2017. Nearly half of the studies (39 of 80) used ML or deep learning, reflecting growing interest in applying advanced computational techniques to analyze the complex data from digital devices.

### Study Population and Research Setting

**FIG. 3:**
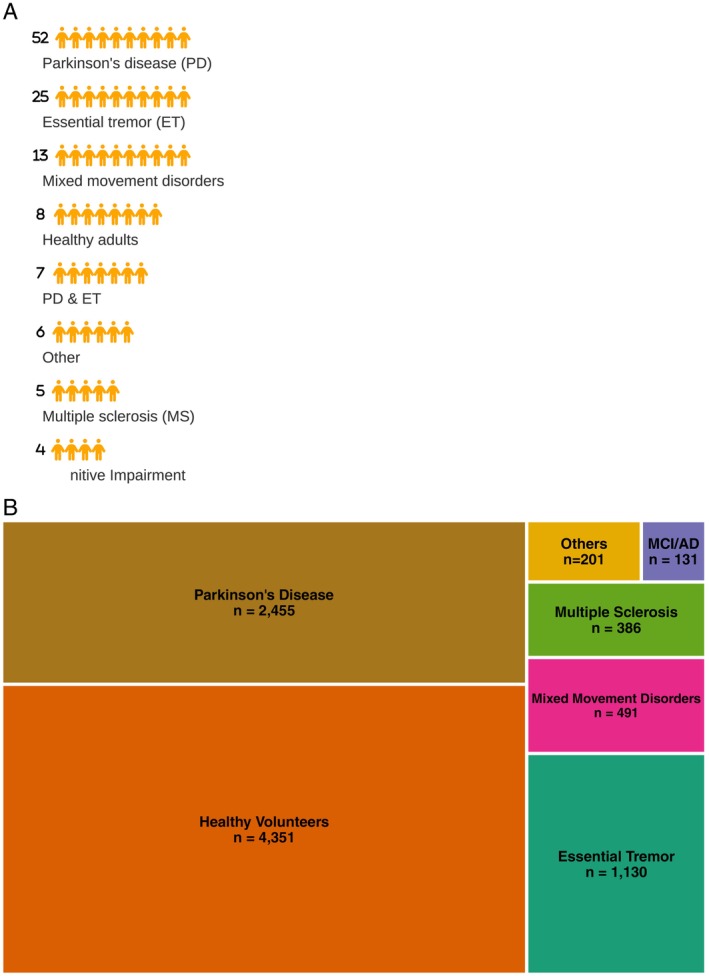
The distribution of studies using the DAST (digitized Archimedes spiral drawing test) across different populations. (**A**) Number of studies by population. (**B**) Total population studied. A small number of studies included participants with overlapping diagnoses (eg, PD [Parkinson's disease] and ET [essential tremor]); for panel A, these studies are counted once per category. Panel B reflects total participants per population, regardless of study overlap.

A significant proportion of the selected studies focus on individuals with PD (n = 52) and ET (n = 25) (Fig. 3A). Other tremor types investigated include kinetic tremor (n = 4),^26^, ^27^, ^28^, ^29^ action tremor (n = 3),^30^, ^31^, ^32^ functional tremor (n = 1),^33^ or physiologic tremor (n = 4).^26^, ^34^, ^35^, ^36^ Additionally, the DAST has been applied to neurological conditions such as multiple sclerosis (MS) (n = 5),^11^, ^37^, ^38^, ^39^, ^40^ ataxia (n = 3),^41^, ^42^, ^43^ dystonia (n = 2),^33^, ^44^ dyskinesia (n = 2),^10^, ^45^ cerebellar disease (n = 3),^43^, ^46^, ^47^ Charlevoix‐Saguenay disease (n = 1),^41^ galactosemia (n = 1),^48^ Niemann–Pick disease type C (n = 1),^13^ mild cognitive impairment (MCI), and Alzheimer's disease (AD) (n = 4).^12^, ^49^, ^50^, ^51^ Additionally, it has been studied on mental fatigue (n = 2),^14^, ^52^ breast cancer survivors (n = 1),^53^ paralympic athletes (n = 1),^54^ and aging populations (n = 4).^26^, ^34^, ^36^, ^55^

The largest participant group consists of individuals without neurologic disorders, included as either controls or volunteers (n = 4239). Clinical populations include PD (n = 2455); ET (n = 1130); MS (n = 386); mixed movement disorders, such as ataxia, bradykinesia, dystonia (n = 491), AD/MCI (n = 131), and others (n = 201) (Fig. 3B).

Most studies (n = 66, 55.0%) were conducted in clinical research settings, including assessment and treatment of patient participants in hospitals, outpatient clinics, or rehabilitation centers. In contrast, 36.6% (n = 44) took place in laboratory‐based settings, typically within academic institutions, involving healthy volunteers under controlled experimental conditions. Nearly half of these lab‐based studies (n = 21, 47.0%) used publicly available datasets. Ten studies were conducted in home or community settings.^37^, ^55^, ^56^, ^57^, ^58^, ^59^, ^60^, ^61^, ^62^, ^63^ Several of these reported high user compliances and showed that spiral data collected remotely correlated well with clinical ratings (eg, Unified Parkinson's Disease Rating Scale UPDRS, The Parkinson's Disease Questionnaire (PDQ‐39)), with intraclass correlation coefficients (ICC) up to 0.87.^59^, ^61^ These findings support the feasibility and potential validity of using spiral drawing assessments in unsupervised, at‐home contexts, although some studies reported challenges like missing follow‐up visits or limited validation in remote setting.^37^, ^56^

For study design, observational cross‐sectional studies were the most common (n = 80, 66.6%). Longitudinal or pre‐/post‐intervention studies accounted for 15.8% (n = 19), whereas experimental studies with model development accounted for 17.5% (n = 21). Further details of all included studies are provided in Table S4.

### Spiral Acquisition and Drawing Task

Various devices and methods were used to capture spiral data (Table S5). Commercial tablets were the most used device, reported in 80 studies (66.6%). The Wacom (Wacom Technology Corporation) is the most frequently used (n = 41). Other brands of tablets were mentioned in 16 studies and iPads in 6 studies, and 17 studies did not specify the tablet type. In some studies, paper‐based drawings were digitized by scanning (n = 11) or photographing them (n = 1). Although smartphones have smaller screens, they were used in 8 studies. Eleven studies did not provide any information on the devices used.

**FIG. 4:**
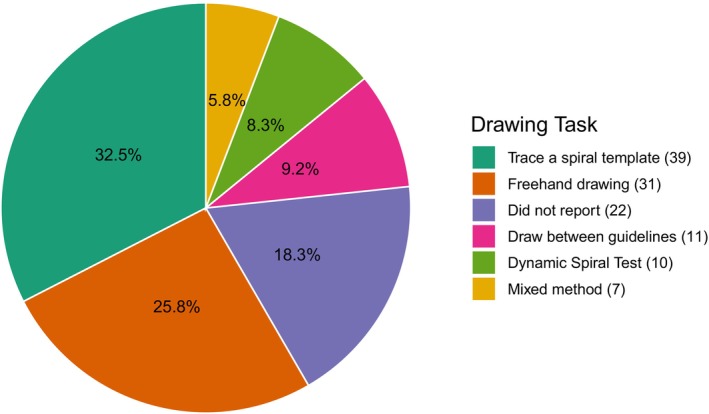
Proportion of reported drawing tasks.

About one‐third of studies (32.5%) used a predefined spiral template for tracing. However, there was considerable variation in task design, such as spiral drawing direction (inward/outward spirals),^26^, ^34^, ^45^ size (large or small), shape (eg, circle, square, octagon), tracing guidance (stationary or fixed speed),^45^, ^54^, ^64^, ^65^, ^66^, ^67^ and input method (finger, pen, or stylus).^37^, ^41^, ^57^, ^58^, ^68^, ^69^, ^70^, ^71^, ^72^, ^73^ Other tasks included freehand drawing (n = 31), drawing between template lines (n = 11), and dynamic drawing (n = 10), where participants follow a blinking spiral. However, 22 studies (18.3%) did not describe how the drawing tasks were administered, making it difficult to compare methods across studies (Fig. 4).

Of 120 studies, 76 (63.3%) did not specify speed and accuracy instructions. Among those that did, 29 studies instructed participants to draw at a “natural speed” with accuracy, and 9 emphasized both accuracy and speed (eg, “as accurately and as fast as possible”).^37^, ^51^, ^53^, ^55^, ^60^, ^61^, ^72^, ^74^, ^75^ Two studies specifically emphasized speed, providing instructions such as “complete the drawing within 10 seconds”^12^, ^67^; 52 of 120 (43%) studies did not mention which hand was used to draw; 36 studies (30%) included both dominant and nondominant hands, 31 studies (25.8%) used only the dominant hand, and 1 study analyzed severe hand or dominant hand. Among the 70 (58%) studies that report total drawing counts, the number of drawings per participant typically ranged from 1 (reported in 11.6% of total studies) to 20 (also in 11.6% of total studies). Further details of drawing task, device, and analysis methods of all included studies are provided in Table S6.

### Key Measures and Analysis Method

A wide variety of drawing features and measures have been used to analyze the digital spirals. Most studies used multiple measures, including spatial, temporal, kinematic, and tremor‐related features (Table 1). Commonly reported outputs included *x*–*y* coordinate data, drawing time, drawing length, number of pen lifts, and pen pressure. These outputs (raw data) were usually transformed into derived measures, such as speed, velocity, and deviation from an ideal path. Tremor‐specific features like tremor frequency, amplitude, and spectral power were often analyzed using fast Fourier transform and spectral analysis.

**TABLE 1:** Frequently used DAST metrics and features

Category	Description	Examples	Applications
Spatial	Physical characteristics of the spiral.	Position (*x*–*y* coordinates), path length, linearity, deviation from an ideal template (error metrics), line smoothness.	Assesses spatial accuracy, tremor amplitude, motor coordination, and deviations caused by fine motor impairments.
Temporal	Timing‐related features.	Total task duration, segment‐specific time, variability in timing, and pauses (start/stop instances).	Evaluates motor planning, execution rhythm, bradykinesia, and timing irregularities associated with motor disorders.
Kinematic	Movement dynamics, including motion smoothness and oscillations.	Overall drawing speed, changes in velocity, acceleration, angular speed around curves, oscillations in the frequency domain, movement jerkiness.	Detects and characterizes tremors, quantifies motor speed, assesses smoothness of movement, and identifies fatigue or movement irregularities.
Pressure	The pressure exerted by a pen, stylus, or finger during the drawing task.	Force applied, pressure variability.	Measures grip strength, motor control, consistency, and fine motor impairments involving reduced or irregular pressure control.
Tremor	Characteristics of tremors.	Tremor amplitude, root mean square displacement, power spectral density, tremor frequency (Fourier transform or fast Fourier transform).	Diagnoses and quantifies tremor types (eg, PD or ET) and assesses severity.
Image based	Features extracted directly from spiral images.	Geometric attributes (symmetry, angles), shape features (edge detection, contours), pixel intensity, deep learning representations (via CNN/Deep Learning (DL) models).	Automates classification of motor impairments, analyzes spiral shapes/patterns, and integrates with AI/ML for diagnostic tools.
Combined	Composite metrics derived from multiple categories or selected features optimized for machine learning models.	Degree of severity, approximate entropy, temporal irregularity score, mean spiral score, multimodal metrics (eg, combining spatial, temporal, and kinematic data), feature selection (eg, ML based).	Holistic assessment of motor function, data integration for diagnostic accuracy, and enhancing research reproducibility.

Several studies created composite scores for a more interpretable assessment of motor function. The most reported composite metric (n = 8) was degree of severity (DoS), which is a weighted, computational score of spiral execution that integrates spiral shape and line smoothness.^8^, ^35^, ^44^, ^73^, ^76^, ^77^, ^78^, ^79^ ML studies extracted a wide range of features, often in the hundreds. However, many of them were highly correlated due to transformations of the same underlying raw data.^80^, ^81^

Analytical approaches varied based on study objectives. Traditional methods such as group comparisons, correlation analyses, and regression models were the most common (65.8%, 79 of 120). ML techniques were used in 28 studies, often applying feature selection algorithms for classification or prediction. Deep learning models, particularly convolutional neural networks (CNN), were used in 19 studies to classify motor impairments from spiral images, usually without predefined kinematic or tremor‐related features.

### Measurement Properties

Of the 120 reviewed studies, 52 (43.3%) investigated at least 1 of the key measurement properties: reliability, validity, or responsiveness. Table S7 presents these key properties, as defined by COSMIN. Most of these studies focused on PD and ET, whereas fewer assessed other clinical populations. In those studies using ML/AI methods, only 9 of 41 studies addressed the evaluation of measurement properties.^16^, ^18^, ^46^, ^60^, ^75^, ^82^, ^83^, ^84^, ^85^ To reflect this imbalance and better evaluate the strength of the evidence base, the following results are grouped by condition type.

### Reliability

#### Reliability in PD and/or ET

Fourteen studies reported test–retest reliability using Pearson's correlation, coefficient of variation, or Bland–Altman analysis (Table S8).^10^, ^11^, ^18^, ^27^, ^28^, ^30^, ^54^, ^60^, ^62^, ^66^, ^72^, ^74^, ^75^, ^86^, ^87^ Of these, 11 focused on PD and/or ET, with reproducibility ICCs ranging from 0.74 to 0.97.^27^, ^30^, ^66^, ^72^, ^74^, ^75^, ^88^ Four studies compared inter‐rater reliability between digital measures and visual ratings, reporting a weighted kappa coefficient of 0.65 to 0.95.^18^, ^30^, ^60^, ^62^ For example, Legrand et al. reported excellent inter‐rater reliability for 4 computerized scores, with an overall ICC of 0.91.^30^ Similarly, Holcomb et al. developed a CNN‐based model (electronic Archimedes spiral neural network) that achieved near‐perfect agreement with expert ratings (κ = 0.93–0.95) using the Bain and Findley Spirography Rating Scale. These studies highlighted the reliability of device‐driven assessments in replicating clinical judgments.^18^ In contrast, Memedi et al. found lower inter‐rater reliability, with a weighted kappa of 0.65 between a multilayer perceptron classifier and 4 human raters, although this exceeded the agreement among human raters themselves (range: 0.23–0.63).^60^ Measurement error was reported in 2 studies.^66^, ^72^ Elble and Ellenbogen (2017) calculated the minimum detectable change (MDC) for both digitized spiral metrics and visual ratings in ET, with MDC values of 51 and 67% of baseline values depending on transformation methods, respectively.^66^ This study also emphasized that clinical scales such as the Fahn–Tolosa–Marin (FTM) rating are ordinal and nonlinear, and presented a regression‐based approach to convert FTM scores to amplitude estimates using a log‐transformed scale. Schallert et al. calculated the 95% of minimum detectable differences for various spiral and tapping tasks, reporting ranging from 14% to 38% for accuracy‐based metrics.^72^

#### Reliability in Other Conditions

Only 3 studies assessed reliability outside of PD/ET populations, examining groups with MS, paralympic athletes, or patients with other neurological disorders.^11^, ^54^, ^72^ These studies used similar methods (test–retest correlation or agreement statistics) but yielded more variable results, with ICC ranges from 0.2 to 0.95 based on different metrics (see Table S8). Although they suggest potential for broader use of the DAST, evidence on reproducibility remains preliminary. Measurement error metrics were reported in 1 study involving patients with action tremor. Legrand et al. quantified the standard error of measurement across multiple spiral‐derived indices using repeated trials and found that the variability in these computerized measures was lower than that observed in expert visual ratings.^30^

### Validity

#### Validity in PD and/or ET

Validity was assessed in 45 studies across various clinical populations (Table S9). Twelve studies (27.3%) each focused on PD^10^, ^29^, ^45^, ^60^, ^62^, ^65^, ^74^, ^75^, ^78^, ^79^, ^85^, ^89^ and ET,^7^, ^28^, ^30^, ^46^, ^66^, ^77^, ^86^, ^87^, ^88^, ^90^, ^91^, ^92^ with 3 studies evaluating both.^27^, ^56^, ^93^

Criterion validity was assessed in 12 PD/ET studies, showing strong correlations with gold‐standard measures like accelerometry, clinical tremor scales, and visual ratings.^7^, ^27^, ^28^, ^56^, ^62^, ^65^, ^66^, ^86^, ^87^, ^90^, ^94^, ^95^ For example, DAST measures such as tremor amplitude, frequency, and velocity strongly correlate with clinical tremor scales, including FTM tremor ratings (r = 0.94), Bain and Findley ratings (R^2^ = 88.9%, *P* < 0.0001), and accelerometry (r = 0.7–0.9).^7^, ^56^ Elble and Ellenbogen also demonstrated a strong logarithmic relationship between tablet‐measured tremor amplitude and clinical FTM spiral ratings, reinforcing earlier findings by Haubenberger et al. showing r > 0.9 log‐linear correlation between amplitude and visual severity scores.^66^, ^88^. These findings highlight the potential for digital spiral metrics to reflect symptom severity in a clinically interpretable, albeit nonlinear, scale.^66^, ^86^

Convergent validity assessed in 23 studies generally showed moderate to strong relationships between spiral metrics and established clinical tools (eg, UPDRS: r ≈ 0.4–0.6, Bain Tremor Rating Scale: r ≈ 0.5–0.8, the Scale for the Assessment and Rating of Ataxia: r ≈ 0.6).^10^, ^11^, ^26^, ^30^, ^34^, ^37^, ^39^, ^42^, ^43^, ^44^, ^45^, ^51^, ^55^, ^60^, ^62^, ^74^, ^75^, ^79^, ^85^, ^87^, ^91^, ^94^ DAST metrics also correlated with motor performance tests, such as the 9‐hole peg test and box‐and‐block test (r ≈ 0.4–0.8).

Discriminant or known‐group validity was assessed in 33 studies to evaluate the ability of the DAST measures to distinguish between clinical and nonclinical populations.^11^, ^12^, ^13^, ^29^, ^33^, ^37^, ^41^, ^42^, ^43^, ^44^, ^46^, ^51^, ^53^, ^55^, ^60^, ^62^, ^65^, ^72^, ^77^, ^78^, ^85^, ^86^, ^89^, ^91^, ^92^, ^93^, ^94^, ^95^ In PD, spiral metrics such as DoS, smoothness, and entropy demonstrated moderate correlations with UPDRS III motor subscores (eg, r = 0.50–0.64).^10^, ^45^, ^62^, ^75^, ^79^, ^85^ Other features, including tremor frequency, spectral characteristics, and force, have also shown high discriminative validity in differentiating PD patients from controls (*P* < 0.001 for multiple indices).^10^, ^29^, ^60^, ^65^, ^74^, ^78^, ^89^, ^93^ However, findings in early‐stage PD are still mixed. For example, DoS and spiral width variability index distinguish early PD patients from controls (*P* < 0.05), whereas temporal irregularity score does not (*P* = 0.07).^74^, ^78^

#### Validity in Other Clinical Populations

Eighteen studies assessed validity in other clinical populations. These included MS (n = 3),^37^, ^39^, ^65^ cognitive impairment (n = 2),^12^, ^70^ mixed movement disorders (n = 7),^33^, ^41^, ^42^, ^43^, ^44^, ^72^, ^94^ Niemann–Pick disease type C (n = 1),^13^ galactosemia (n = 1),^48^ breast cancer survivors (n = 1),^53^ and adults without neurologic disorders (n = 3).^26^, ^34^, ^55^

In MS, spiral metrics like drawing time and speed variability correlate with the 9‐hole peg test completion times (r = 0.55, *P* = 0.03) and show strong known‐group validity, with segment rate analysis achieving sensitivity as high as 84.6% compared to manual scoring (61.5%).^11^, ^37^, ^39^

In aging population, Hoogendam et al. (2014) linked older age to poorer spiral‐drawing performance (*P* < 0.001) and showed significant correlations with brain volumes on magnetic resonance imaging (MRI) scans (*P* < 0.05),^55^, ^86^ and Koppelmans et al. reported that metrics such as movement time and speed variability failed to differentiate healthy control, MCI, and AD groups or correlate with AD biomarkers, suggesting limited sensitivity to cognitive changes.^51^ Similarly, Fujiwara et al. found that standalone spiral metrics were less reliable in differentiating MCI and AD patients from nonpatients.^12^ However, when cognitive challenges, such as counting color changes, were incorporated into the task, the dual‐task method significantly enhanced drawing performance differentiation between MCI and non‐MCI patients, achieving 90% sensitivity and 75% specificity.^12^

### Responsiveness

#### Responsiveness in PD and/or ET

Six studies examined the DAST's ability to detect motor changes post‐intervention.^56^, ^74^, ^75^, ^86^, ^89^, ^96^ The DAST demonstrates strong responsiveness to treatment‐related motor changes across various interventions, including levodopa (l‐dopa), ethanol, MR‐guided focused ultrasound thalamotomy, and deep brain stimulation.^56^, ^74^, ^75^, ^86^, ^89^, ^96^ Key findings and clinical applications are summarized in Table S10.

#### Responsiveness in Other Conditions

No studies to date have evaluated responsiveness in non‐PD/ET populations, highlighting a major gap in the evidence base for broader clinical applications.

## Conclusion

### Summary of Evidence

This scoping review maps how the DAST has been used to assess fine motor function in adults. The DAST demonstrates strong validity and reliability in PD and ET, but evidence for other conditions remains limited. Although digital tools can detect subtle motor changes, differences in devices, task designs, and how studies are reported make it difficult to standardize the approach. The following sections discuss these aspects within the population/concept/context framework, providing a structured overview of its applications, measurement properties, and clinical utility.

### Population

Whereas most studies (70.8%, 85 of 120) have focused on PD and ET, the DAST has been used in a wider range of populations, including conditions not primarily associated with movement disorders, such as breast cancer survivors, patients with MCI or AD, aging individuals, paralympic athletes, and individuals experiencing mental fatigue.^12^, ^14^, ^26^, ^36^, ^49^, ^51^, ^52^, ^53^, ^55^ Currently, studies on responsiveness and construct validity are limited to PD and ET.

### Context

The results reveal significant variability in research methods and reporting, posing major challenges for standardization and cross‐study comparisons. Devices used to collect data ranged from high‐resolution tablets to smartphones, scanned paper drawings, and digital pens. Each device differs in resolution, sensitivity, and input methods, which can influence drawing accuracy and measurement quality.^57^, ^68^, ^73^ Task design also varies. Whereas most studies used a stylus or pen for drawing, some used finger input.^37^, ^41^, ^57^, ^68^, ^70^, ^72^ Additionally, testing procedures varied. Some studies instructed participants to prioritize drawing speed, whereas others emphasized drawing accuracy. A major limitation is that 63.3% (76 of 120) of studies did not report detailed task instructions, making it impossible to fully evaluate and interpret results. Other factors, such as hand use, task design, and the number of repeated trials, are inconsistently reported, despite their impact on motor assessments.^69^

The DAST's strength lies in its ability to address the heterogeneity of motor symptoms through various measures, including raw data (eg, drawing time and length), derived metrics (eg, smoothness and velocity), composite scores (eg, DoS), and features extracted by ML/AI techniques. This flexibility allows for a comprehensive assessment of fine motor function across conditions. However, the absence of standardized protocols for processing and analyzing digitized drawings introduces variability and hampers reproducibility. Operator‐dependent factors, such as clip‐mark placement, time‐series selection, spiral start‐ and endpoints, and data processing methods, can significantly influence outcomes, yet only a few studies address these issues.^15^, ^30^, ^86^, ^97^

Despite the lack of methodological transparency in some AI/ML studies, ML models generally require large numbers of samples to effectively learn patterns and achieve robust generalization for clinical prediction. Depending on the model and task complexity, limited data may constrain model performance and increase the risk of overfitting. Most studies include fewer than 100 participants, with many having 20 or fewer drawings per participant, potentially undermining the reliability of ML methods in this specific context.^98^ To enhance reproducibility and practical application in both clinical and research settings, there is a need for more robust methodologies to select the most meaningful spiral metrics and establish standardized protocols for data processing.^99^

### Content

A key focus of this review is to evaluate the measurement properties of the DAST, including validity, reliability, and responsiveness. Among the 52 studies reporting at least 1 of these properties, considerable variability was observed both within and across studies.

### Reliability

Among the 14 studies reporting test–retest reliability, most demonstrated moderate to strong reliability, with ICC values typically exceeding 0.75. However, some studies reported lower‐than‐expected reliability, especially in systems that combine multiple motor tasks or use complex data processing methods.^72^, ^75^ Although digital assessments are expected to be more consistent than human ratings, these findings suggest that the method of data collection and analysis can impact reliability, emphasizing the need for clearer guidelines and more standardized methods.^75^

Digital assessments address significant limitations of traditional visual ratings by reducing rater variability. For example, Haubenberger et al. demonstrated this advantage by showing improved reproducibility using digital methods.^86^ Using a single visual rater, 28 patients with ET were required to document a 50% reduction in tremor amplitude, whereas only 9 patients were needed with computerized methods (power: 95%, α: 0.05).^86^ However, despite their superior precision, digital methods are constrained by the natural variability in tremor amplitude over time. For example, Elble and Ellenbogen reported comparable MDC between digital methods and traditional Fahn–Tolosa–Marin ratings of Archimedes spirals in assessing ET.^66^ This finding highlights an important limitation: although tablets and other digital tools measure variability with high accuracy, any observed change must exceed random variability to be considered statistically significant. As a result, even highly precise digital tools may not consistently outperform traditional methods in detecting meaningful changes. Rigorous validation, standardization, and careful consideration of clinical relevance are needed to integrate digital approaches into practice.

### Validity

Numerous studies support the validity of the DAST, yet its sensitivity and generalizability remain areas of debate. Although digital spiral metrics generally correlate well with visual ratings, their strength varies depending on task design, selected metrics, and the reliability of visual ratings. For example, Lin et al. found that tracing a spiral template showed stronger correlations with visual ratings (r = 0.97 and 0.95) compared to freehand drawing (r = 0.69).^27^ The study suggested that freehand drawing, characterized by unevenly spaced spirals and varying speeds, introduces complexities that visual ratings struggle to fully capture. Interestingly, these limitations in visual ratings, rather than deficiencies in digital metrics, may explain the weaker correlations, underscoring the validity of digital metrics despite such challenges.

The DAST has demonstrated strong validity in differentiating movement disorders, particularly in PD and ET, where spiral metrics correlate with standard clinical scales and objective tremor measures. However, its effectiveness in early‐stage disease depends on the selected metrics and task design.^74^, ^78^ Beyond movement disorders, evidence for DAST validity in broader constructs and populations is limited. For instance, the UPDRS total score, which includes nonmotor components such as mood, behavior, and activities of daily living, shows weaker or more inconsistent correlations with DAST metrics compared to motor subscores.^42^, ^79^ Furthermore, evidence supporting the validity of the DAST in older adults and samples with cognitive impairment is sparse, despite the well‐documented impact of aging on fine motor function.^100^, ^101^, ^102^

Overall, the evidence suggests that although DAST is well established for motor impairments, its role in nonmotor and cognitive assessments remains uncertain. Future research should refine spiral measures to better capture condition‐specific motor symptoms and expand validation in broader populations to enhance their clinical applicability.

### Responsiveness

The current evidence demonstrates the DAST's potential as a sensitive tool for detecting treatment‐related changes in motor function in PD and ET. Metrics like drawing velocity and tremor intensity consistently reflect improvements after interventions such as l‐dopa administration, ethanol treatment, MR‐guided focused ultrasound thalamotomy, and deep brain stimulation.^56^, ^74^, ^75^, ^86^, ^89^, ^96^

However, research on responsiveness remains limited, with only a small number of studies evaluating this measurement property. The lack of evidence across diverse interventions, patient populations, and motor conditions highlights a major gap. Further studies are needed to validate the DAST's responsiveness in broader clinical contexts and to ensure its utility as a reliable outcome measure for tracking therapeutic efficacy.

### Future Directions

This review highlights the DAST's potential as a precise, objective, and noninvasive outcome measurement tool for both clinical trials and routine care. However, several critical gaps should be addressed to maximize its utility and clinical relevance:

1. **Expanding applications beyond PD and ET:** although DAST has primarily been used to assess motor function in these conditions, its potential extends to a broader range of clinical applications. However, its reliability outside of PD and ET remains largely unexamined. Systematic evaluation across varying populations is needed to establish its broader relevance and support its adoption as a standard tool for fine motor assessment in research and clinical practice. In addition, population‐level studies in adults without neurological disorders could help establish normative reference values across the lifespan and enhance our understanding of the natural history of movement disorders and the neurophysiological basis of fine motor function.
2. **Standardizing data collection, analysis, and reporting:** a key barrier to broader DAST adoption is the lack of standardized protocols. To support reliability and validity assessment, COSMIN guidelines emphasize the importance of clearly reporting how data are collected and handled (eg, how multiple trials were averaged, how missing data were treated). Researchers should clearly report key details about how the DAST was conducted, including device specifications (eg, tablet model, resolution, sampling rate), input method (stylus or finger), and task instructions (eg, hand used, number of trials), and how data were analyzed. Without these standardized reporting practices, DAST metrics risk becoming difficult to interpret and clinically irrelevant. Establishing these guidelines will enhance the DAST's reliability and utility in both research and clinical applications.
3. **Strengthening the validation and reporting of ML/AI applications:** most ML/AI studies using the DAST focus on algorithm development and diagnostic accuracy, with little emphasis on their use as measurement tools. Although these methods improve feature extraction and automate scoring, their reliability and clinical utility remain largely unvalidated. To establish DAST‐derived AI metrics as meaningful outcome measures, future research must go beyond classification accuracy and rigorously assess measurement properties. Ensuring AI‐derived scores are interpretable and suitable for longitudinal tracking is essential for their use in clinical and research settings. Equally important is adopting standardized reporting practices to improve reproducibility and clinical applicability. Guidelines like MINimum Information for Medical AI Reporting^103^ and Minimum Information about Clinical Artificial Intelligence Modeling^104^ should be followed to document data collection, cohort characteristics, preprocessing, and model development, which are critical for interpreting AI‐derived DAST scores.
4. **Evaluating responsiveness to clinical treatments or therapies:** a key area for future research is assessing the DAST's responsiveness to therapeutic interventions across diverse patient populations. Establishing its ability to detect meaningful changes in motor function over time is crucial for its use in therapeutic monitoring. Expanding studies on responsiveness will strengthen its application as a reliable outcome measurement tool in both clinical practice and research.
5. **Enhancing clinical relevance:** although the DAST provides many valuable metrics for research, the sheer volume of data can reduce its clinical usability. Unlike clinical ratings, which are inherently interpretable despite potential reliability issues, DAST measures and metrics require additional context to convey clinical meaning. To improve its practical value, future efforts should refine and prioritize metrics that are most relevant to specific conditions and clinical decision‐making. Together with refined metrics, contextual workflow integration of automated AI/ML evaluation tools could further increase the clinical value of the DAST.

## Limitations

This scoping review has several limitations. First, it includes only peer‐reviewed publications, excluding unpublished studies and gray literature, which may limit the breadth of evidence. Despite a structured framework and comprehensive search, some relevant studies may have been missed, especially due to the English‐only restriction, which reduced the number of eligible studies. Additionally, methodological quality was not assessed, meaning sample size was not a criterion for exclusion, and studies with very small participant numbers were included. Although this aligns with the broad scope of a scoping review, it also means studies with limited statistical power were considered alongside larger studies. Due to the large number of included studies, assessing methodological quality could have strengthened interpretations and provided deeper insights.

## Author Roles

(1) Research project: A. Conception, B. Organization, C. Execution; (2) Statistical analysis: A. Design, B. Execution, C. Review and critique; (3) Manuscript: A. Writing of the first draft, B. Review and critique.

S.W.: 1A, 1B, 1C, 2A, 2B, 3A, 3B

T.S.: 1B, 1C, 2C, 3B

D.M.: 1B, 1C, 2C, 3B

M.B.: 1A, 1B, 1C, 3B

A.W.H.: 1A, 2C, 3B

## Disclosures

**Ethical Compliance Statement**: The authors confirm that the approval of an institutional review board or patient consent was not required for this work. For this work, no informed consent was obtained. They confirm that they have read the journal's position on issues involved in ethical publication and affirm that this work is consistent with those guidelines.

**Funding Sources and Conflicts of Interest**: The authors declare that there are no funding sources or conflicts of interest relevant to this work.

**Financial Disclosures for the Previous 12 Months**: S.W. was employed by Applied Therapeutics, Inc. D.M. is the owner and employee of Cerebral Innovations, Inc, which provides an FDA (Food and Drug Administration)–adhering DAST and consulting services to pharmaceutical and biotech companies. T.S. M.B., and A.W.H. have no additional disclosures to report.

## Supporting information


**Table S1.** Search method.

**Table S2.** Excluded papers and reason for exclusion.

**Table S3.** Data extraction table.

**Table S4.** Country, setting, and population for all included studies.

**Table S5.** Device used in the studies of spiral drawing test.

**Table S6.** Drawing task, device, and analysis method for all included studies.

**Table S7.** COSMIN (Consensus‐Based Standards for the Selection of Health Measurement Instruments) measurement properties and application to DAST (digitized Archimedes spiral drawing test).

**Table S8.** Studies that measure reliability.

**Table S9.** Studies that measure validity.

**Table S10.** Studies that measure responsiveness.

## Data Availability

The data that support the findings of this study are available on request from the corresponding author.
