# Supplementary material for: Clinical Applications and Measurement Properties of the Digitized Archimedes Spiral Drawing Test: A Scoping Review
Source: Mov Disord Clin Pract. 2025 Aug 7;12(11):1742–55. doi: 10.1002/mdc3.70278 (PMC12625189; doi:10.1002/mdc3.70278)
Supplement: Supplementary file 1 — Table S1. Search method. [file MDC3-12-1742-s004.docx]

## Table S1. Search Method

The search strategy is designed to capture studies on the Digitized Archimedes Spiral Drawing Test (DAST) across various applications and measurement properties. The following terms include both keywords and Medical Subject Headings (MeSH) terms, using Boolean logic, truncation, and wildcards to maximize the retrieval of relevant articles. No language or date limits have been applied to ensure a comprehensive search.

(Tablet* OR "Computers, handheld"[MeSH] OR Touchscreen* OR Handheld-computer* OR smartphones[MeSH] OR electronic* OR digitized OR digital OR "Diagnosis, Computer-Assisted"[Mesh]) AND (spiral-drawing* OR spirograph* OR spiral-test* OR archimedes-spiral* OR spiral-pattern OR spiral-analys*)

Name of database: MEDLINE (PubMed), Embase (Elsevier), CINAHL (EBSCO), PsycINFO (EBSCO), Scopus (Elsevier), and Web of Science (Clarivate Analytics)

| **Database** | **Vendor** | **Date Searched** | **# of Results** | **Duplicates Removed** | **# of Unique Results** |
| --- | --- | --- | --- | --- | --- |
| MEDLINE | PubMed | 11/1/2024 | 151 | 0 | 151 |
| Embase | Elsevier | 11/1/2024 | 631 | 153 | 478 |
| CINAHL | EbscoHost | 11/1/2024 | 17 | 17 | 0 |
| PsycINFO | EbscoHost | 11/1/2024 | 16 | 14 | 2 |
| Scopus | Elsevier | 11/1/2024 | 391 | 236 | 155 |
| Web of Science | Clarivate | 11/1/2024 | 201 | 157 | 44 |
| Total |  |  | 1407 | 577 | 830 |
