# Supplementary material for: Clinical Applications and Measurement Properties of the Digitized Archimedes Spiral Drawing Test: A Scoping Review
Source: Mov Disord Clin Pract. 2025 Aug 7;12(11):1742–55. doi: 10.1002/mdc3.70278 (PMC12625189; doi:10.1002/mdc3.70278)
Supplement: Supplementary file 2 — Table S2. Excluded papers and reason for exclusion. [file MDC3-12-1742-s009.docx]

## Table S2. Excluded Papers and Reason for Exclusion

| **First Author** | **Publication Title** | **Reason for Exclusion** | **Detail Reason** |
| --- | --- | --- | --- |
| Accardo A | Kinematic analysis of tremor - biomed 2010. Biomed Sci Instrum | Records not Retrieved | Records not Retrieved |
| Aghanavesi S | Measuring temporal irregularity in spiral drawings of patients with Parkinson's disease | No full text | Abstract only |
| Akano E | Fahn-Tolosa-Marin scale, digitizing tablet and accelerometry have comparable minimum detectable change | No full text | Abstract only |
| Almeida MFS | Investigation of changes in kinetic tremor through analysis of hand-drawing movements: Differences between physiological and essential tremors | Wrong publication type | Case study |
| Augustyn M. | Analysis of changes in pen pressure while drawing Archimedes' spiral using a graphics tablet by healthy people and patients with Parkinson's disease | Records not Retrieved | Records not Retrieved |
| Bajaj NP | Can spiral analysis predict the FP-CIT SPECT scan result in tremulous patients? Movement Disorders | Wrong method | Wrong Scoring Method - Visual scoring only |
| Banaszkiewicz K | An assessment of bradykinesia by means of computerized analysis of spiral drawing on the graphic digitizing tablet | No full text | Abstract only |
| Berki A | Quantifying the effect of subthalamic stimulation on bradykinesia using digitized spiral drawings in Parkinson's disease | No full text | Abstract only |
| Bukowczan S | Differentiation of primary writing tremor and writer's cramp using quantitative tremor assessment by the spectral analysis of spiral drawing on graphic digitizing tablet | No full text | Abstract only |
| Chen KH | Correlation between tremor severity of essential tremor and perpendicular displacement in digital spiral analysis | No full text | Abstract only |
| Chen KH | An optimized formulation of velocity distribution in digital spiral analysis to differentiate essential tremor and Parkinson's disease | No full text | Abstract only |
| Chomiak T | A versatile computational algorithm for time-series data analysis and machine-learning models | No full text | Abstract only |
| Danna J | Computerized analysis of spiral drawing in Parkinson's disease | No full text | Abstract only |
| Elble R | Task force report: Scales for screening and evaluating tremor: Critique and recommendations | Wrong publication type | Review |
| Elble RJ | Using Portable Transducers to Measure Tremor Severity | Wrong publication type | Review |
| Faundez-Zanuy M | Online Handwriting, Signature and Touch Dynamics: Tasks and Potential Applications in the Field of Security and Health | Wrong publication type | Review |
| Fung WKW | "stretched Slinky" Sign: Another Clue to Functional Tremor | Wrong publication type | Case study |
| Gagnon C | Validity and Reliability of Outcome Measures Assessing Dexterity, Coordination, and Upper Limb Strength in Autosomal Recessive Spastic Ataxia of Charlevoix-Saguenay | Wrong method | Wrong Scoring Method - Visual scoring only |
| Geny C | Contribution of different drawing tasks in the assessment of essential tremor | No full text | Abstract only |
| Geny CB | Contribution of different drawing tasks in the assessment of essential tremor | No full text | Abstract only |
| Groznik V | Attribute visualisation for computer-aided diagnosis: A case study | Wrong publication type | Wrong Publication Type - summary of a doctoral thesis |
| Groznik V. | Artificial intelligence methods for modelling tremor mechanisms | Wrong publication type | Case study |
| Gupta H | Spiral drawing in myoclonus | No full text | Abstract only |
| Halder S | Handwriting analysis in Writer's cramp using a digitizing tablet | No full text | Abstract only |
| Hamaguchi M | Characteristics of chronic fatigue syndrome in a Japanese community population: Chronic fatigue syndrome in Japan | Wrong method | Wrong Outcomes - Does Not Include DAST. |
| Haubenberger D | Transducer-based evaluation of tremor | Wrong publication type | Review |
| Haubenberger D | Objective response to ethanol in essential tremor: Results from a standardized ethanol challenge study | No full text | Abstract only |
| Haubenberger D | Computerized essential tremor spirography: Development of a rater-independent outcome measure for clinical trials | No full text | Abstract only |
| Haubenberger D | Digital Spiral Analysis for Objective Quantification of Limb Dysmetria - A Validation Study | No full text | Abstract only |
| Hernandez AB | WHIGET and TETRAS Ratings of Action Tremor in Patients with Essential Tremor: Substantial Association and Agreement | Wrong method | Wrong Scoring Method - Visual scoring only |
| Hess CW | Tremor: Clinical phenomenology and assessment techniques | Wrong publication type | Review |
| Hess CW | Normative data for digitized spiral analysis: Evaluation of age and gender effects | No full text | Abstract only |
| Hollý P | Estimating Disability in Patients with Essential Tremor: Comparison of Tremor Rating Scale, Spiral Drawing, and Accelerometric Tremor Power | Wrong method | Wrong Scoring Method - Visual scoring only |
| Hopfner F | Testing for alcohol sensitivity of tremor amplitude in a large cohort with essential tremor | Wrong method | Wrong Scoring Method - Visual scoring only |
| Jaiswal S | Effect of multiple sessions of transcranial direct current stimulation in Writer's cramp: Does it hold promise? Movement Disorders | No full text | Abstract only |
| Jobbágy Á | Arm tremor assessment in clinical practice | Duplicate | Duplicate |
| Knudsen K | A clinical test for the alcohol sensitivity of essential tremor | Wrong method | Wrong Scoring Method - Visual scoring only |
| Kovács A | Tremor-recording methods and related quantitative tremor parameters in Parkinson's disease | No full text | Abstract only |
| Liu X | Direction of tremor oscillation in spiral drawings reveals proximal versus distal muscle involvement [7] | Wrong publication type | Letter |
| Loesch DZ | Cognitive status correlates of subclinical action tremor in female carriers of FMR1 premutation | Wrong method | Wrong Scoring Method - Visual scoring only |
| Longardner K | Progression of essential tremor over time using digital spirography in patients on stable medication doses compared to untreated patients | No full text | Abstract only |
| Longstaff MG | The influence of motor system degradation on the control of handwriting movements: a dynamical systems analysis | Wrong method | Wrong Outcomes - Does Not Include DAST. |
| Lorenz D | Evaluation of a screening instrument for Essential tremor | Wrong method | Wrong Scoring Method - Visual scoring only |
| Louis ED | The spiral axis: A comparison of unaffected first-degree relatives of essential tremor cases vs | Wrong method | Wrong Scoring Method - Visual scoring only |
| Louis ED | Screening for and estimating the prevalence of essential tremor: A random-digit dialing-based study in the New York metropolitan area | Wrong method | Wrong Scoring Method - Visual scoring only |
| Louis ED | Clinical classification of borderline cases in the family study of essential tremor: An analysis of phenotypic features | Wrong method | Wrong Scoring Method - Visual scoring only |
| Louis ED | Validation of a portable instrument for assessing tremor severity in epidemiologic field studies | Wrong method | Wrong Outcomes - Does Not Include DAST. |
| Luciano MS | Digitized spiral drawing analysis discriminates parkinson disease from control | No full text | Abstract only |
| Luo L | Repeated spiral drawings can distinguish motor learning from tremor changes in patients with essential tremor | No full text | Abstract only |
| Maehrlein W | Automatized processing diagnosis and documentation of spirographic screening test procedures by electronic data processing | Not in English | Not in English |
| Mansur PHG | A review on techniques for tremor recording and quantification | Wrong publication type | Review |
| Masárová L | Assessing handwriting in patients with Parkinson's disease | Not in English | Not in English |
| Memedi M | A method for measuring Parkinson's disease related temporal irregularity in spiral drawings | No full text | Abstract only |
| Memedi M | A web-based system for visualizing upper limb motor performance of Parkinson's disease patients | No full text | Abstract only |
| Memedi M | Visualization of spirography-based objective measures in Parkinson's disease | No full text | Abstract only |
| Memedi M | Spiral drawing during self-rated dyskinesia is more impaired than during self-rated off | Wrong method | Wrong Scoring Method - Visual scoring only |
| Miralles F | Quantitative evaluation of the drawing of a spiral on a paper | Wrong publication type | Letter |
| Moughal S | Looking for inconsistency: Combining distraction with spirography for diagnosis of functional tremor | Wrong publication type | Review |
| Nejad HC | Software development of an intelligent Spirography test system for neurological disorder detection and quantification | Records not Retrived | Records not Retrived |
| Ondo WG | Tetras spirals and handwriting samples: Determination of optimal scoring examples | Wrong method | Wrong Scoring Method - Visual scoring only |
| Ortega R | Progression of motor dysfunction in elderly LRRK2 carriers, including prior to phenoconversion to PD | No full text | Abstract only |
| Ortega R | Spiral analysis is a promising biomarker in LRRK2 G2019S carriers | No full text | Abstract only |
| Ortega R | Discriminative validity of digitized spiral analysis in LRRK2 parkinson disease | No full text | Abstract only |
| Ortega RA | Development of biomarker battery to discriminate LRRK2 mutation carriers | No full text | Abstract only |
| Osterholt T | Distinguishing tremor components in essential tremor: A digital spirography study | No full text | Abstract only |
| Pahwa R | An Acute Randomized Controlled Trial of Noninvasive Peripheral Nerve Stimulation in Essential Tremor | Wrong method | Wrong Scoring Method - Visual scoring only |
| Pandey B | Use of digitizing tablet for the tremor evaluation | No full text | Abstract only |
| Papapetropoulos S | Proof-of-concept, double-blind, placebo-controlled study for CX-8998, a state-dependent t-type calcium (TTCC) modulator in essential tremor patients (T-Calm): Efficacy and safety results | No full text | Abstract only |
| Papapetropoulos S | Efficacy result from a phase 2, double-blind, placebo-controlled study of CX-8998 a state-dependent T-type calcium (CAV3) channel modulator in essential tremor patients (T-CALM) | No full text | Abstract only |
| Paschen S | Long-term efficacy of deep brain stimulation for essential tremor: An observer-blinded study | Wrong method | Wrong Scoring Method - Visual scoring only |
| Peshkin A. | The Use of IncobotulinumtoxinA in the Correction of Tremor in Multiple Sclerosis | No full text | Abstract only |
| Pillai L | Influence of levodopa on archimedes spiral drawings in idiopathic parkinson's disease | No full text | Abstract only |
| Radmard S | Spiral analysis evaluates the effects of deep brain stimulation in Parkinson disease and essential tremor | No full text | Abstract only |
| Rajan R | An automated algorithm for detection and quantification of tremor from pen-on-paper Archimedes spirals | No full text | Abstract only |
| Ratliff JB | Digitally captured Archimedes spiral indices correlate with clinical assessment of dystonia severity | No full text | Abstract only |
| Ratliffy J | Digitized spiral analysis differentiates dystonia patients from control | No full text | Abstract only |
| Razjouyan J | RECURRENT MAP ANALYSIS OF DIGITAL SPIRAL DRAWING SIGNAL TO INVESTIGATE FINE MOTOR MOVEMENT IN MULTIPLE SCLEROSIS PATIENTS AND CONTROLS | Records not Retrived | Records not Retrived |
| Rr R. | Minimum detectable change of an automated algorithm derived spiral analysis parameter | No full text | Abstract only |
| Rudzinska M | Sensitivity and specificity of the quantitative computerized tremor analysis of the spiral drawing on the graphic digitizing tablet | No full text | Abstract only |
| Rudzińska M | Quantitative tremor measurement with the computerized analysis of spiral drawing | Records not Retrived | Records not Retrived |
| Sadikov A | , editors | Wrong population | Non-Adult Populations |
| Schneider SA | A study of subtle motor signs in early Parkinson's disease | Wrong method | Wrong Scoring Method - Visual scoring only |
| Schubert R | QUANTIFYING FINE-MOTOR IMPAIRMENT IN ATAXIA: DIGITAL PARAMETERS OF Q-MOTOR SPIRAL DRAWING CORRELATE WITH CLINICAL SEVERITY, FUNCTION & ADLS - LESSONS FOR HD | No full text | Abstract only |
| Schuhmayer N | Improvement of repeated Archimedes spirals in essential tremor: Evidence for a learning effect? Movement Disorders | No full text | Abstract only |
| Schulze M | TOWARDS DIGITAL-MOTOR OUTCOMES FOR LIMB MOVEMENTS IN SPASTIC ATAXIA: A COMPARATIVE VALIDATION OF Q-MOTOR IN ATAXIA AND HEREDITARY SPASTIC PARAPLEGIA - LESSONS LEARNT FOR HD | No full text | Abstract only |
| Stanley K | Digitized Spiral Analysis Distinguishes Parkinson Disease from Normal Controls | No full text | Abstract only |
| Starita S | Characterization of Parkinson's Disease using spectral features of kinetic tremor: correlation of on-line digitized handwriting and classical motor scales | No full text | Abstract only |
| Sternberg EJ | The “head snap”: A subtle clinical feature during the finger-nose-finger maneuver in essential tremor | Wrong method | Wrong Scoring Method - Visual scoring only |
| Striano P | Levetiracetam for cerebellar tremor in multiple sclerosis: An open-label pilot tolerability and efficacy study | Wrong method | Wrong Scoring Method - Visual scoring only |
| Su N | Application of digital cognition and pen-based sketching tasks in a chinese population-based cohort | No full text | Abstract only |
| Szloboda P | Probabilistic tractrography-based connectivity of the active contact region after VIM-DBS for essential tremor | No full text | Abstract only |
| Taylor K | Prasinezumab reduced progression of Parkinson's disease motor features measured by Roche PD Mobile Application v2 sensor features: PASADENA Phase II Part 1 | No full text | Abstract only |
| Thakur M | Automated restricted Boltzmann machine classifier for early diagnosis of Parkinson’s disease using digitized spiral drawings | No full text | Abstract only |
| Thenganatt MA | Distinguishing essential tremor from Parkinson's disease: Bedside tests and laboratory evaluations | Wrong publication type | Review |
| Unger S | Digital Drawing Tools for Assessing Mental Health Conditions - A Scoping Review | Wrong publication type | Review |
| Valálik I | Motion analysis in the assessment of the effectiveness of stereotactic surgery for movement disorders | No full text | Abstract only |
| Van Lier B | Effect of dopaminergic treatment on digital sensor features in phase II PASADENA part 1 in early Parkinson's disease | No full text | Abstract only |
| Vanitha KM | A wearable system to analyse tremors in the presence of external stressors | Wrong method | Wrong Outcomes - Does Not Include DAST. |
| Vimercati SL | Quantitative evaluation of graphic gesture in subjects with Parkinson's disease and in children with learning disabilities | No full text | Abstract only |
| Wang SY | The direction of oscillation in spiral drawings can be used to differentiate distal and proximal arm tremor | Wrong population | Non-Adult Populations |
| Wesseling C | Parkinson's and Alzheimer's diseases in Costa Rica: a feasibility study toward a national screening program | Wrong method | Wrong Scoring Method - Visual scoring only |
| Westin J | A home environment test battery for status assessment in patients with advanced Parkinson's disease | Wrong method | Wrong Outcomes - Does Not Include DAST. |
| Yamada Y | Digital Measures of Drawing Process to Predict Multiple Cognitive and Gait Measures in Older Adults | Wrong method | Wrong Outcomes - Does Not Include DAST. |
| Yoon H | Quantification of Movement Error from Spiral Drawing Test | Wrong population | Non-Adult Populations |
| Zineddine M | A Novel Approach to Parkinson’s Disease Progression Evaluation Using Convolutional Neural Networks | Wrong method | Wrong Outcomes - Does Not Include DAST. |
