# Supplementary material for: Clinical Applications and Measurement Properties of the Digitized Archimedes Spiral Drawing Test: A Scoping Review
Source: Mov Disord Clin Pract. 2025 Aug 7;12(11):1742–55. doi: 10.1002/mdc3.70278 (PMC12625189; doi:10.1002/mdc3.70278)
Supplement: Supplementary file 3 — Table S3. Data extraction table. [file MDC3-12-1742-s005.docx]

## Table S3. Data Extraction Table

| **Data Item** | **Description** |
| --- | --- |
| **Study Population and Research Context** | |
| First Author | Last Name of the first authors of the study |
| Year of Publication | Year in which the study was published |
| Country/Region | Geographic location(s) where the study was conducted |
| Setting | Description of the clinical or research setting (e.g., clinical, at home etc.) |
| Study Type | Type of study (e.g., observational study, clinical trial) |
| Clinical Population | Specific populations or conditions studied (e.g., Essential Tremor, Parkinson’s Disease, healthy controls) |
| Sample Size | Number of participants included in the study |
| Age Range | Age range of participants included |
| **Spiral Acquisition, Key Metrics, and Analysis Method** | |
| Device used | Details of the device or software used for digitizing the DAST (e.g., graphic tablet, smartphone) |
| Drawing task | Description of how drawing task was conducted in the study (e.g., Tracing, draw between lines, freehand drawing) |
| Drawing Instruction | Detail on speed and accuracy instruction |
| No. of Drawing collected | Total number of drawings collect per participant |
| Hand Analyzed | Details of the dominant or non-dominant hand analyzed |
| Metrics Analyzed | Metrics obtained from DAST (e.g., tremor amplitude, frequency, movement variability) |
| Analysis Methods | Analysis techniques or models used (e.g., statistical methods, AI-based scoring) |
| **Measurement Properties Assessed** | |
| Validity | Type of validity assessed (e.g., construct validity, criterion validity) |
| Reliability | Type of reliability assessed (e.g., test-retest reliability, inter-rater reliability) and key findings |
| Responsiveness | Any responsiveness measures (e.g., changes over time in response to intervention) and key findings |
| **Reporting** | |
| Key Results | Summary of main results related to DAST’s measurement properties |
| Clinical Utility and Implications | Summary of the key takeaways and the clinical utility of the study findings |
