# Supplementary material for: Clinical Applications and Measurement Properties of the Digitized Archimedes Spiral Drawing Test: A Scoping Review
Source: Mov Disord Clin Pract. 2025 Aug 7;12(11):1742–55. doi: 10.1002/mdc3.70278 (PMC12625189; doi:10.1002/mdc3.70278)
Supplement: Supplementary file 4 — Table S4. Country, setting, and population for all included studies. [file MDC3-12-1742-s008.docx]

## Table S4. Country, Setting, and Population for All Included Studies

| **First Author (Year)** | **Country** | **Setting** | **Study Design** | **Population** | **Sample Size** |
| --- | --- | --- | --- | --- | --- |
| Aghanavesi S et al, (2017)^77^ | Sweden | Clinical Research | Longitudinal/pre &post-intervention Study | PD | PD: 19; HC: 22 |
| Aghanavesi S et al, (2017)^78^ | Sweden | Clinical Research | Longitudinal/pre &post-intervention Study | PD | PD: 19; HC: 22 |
| Al-Yousef N et al, (2020)^82^ | Saudi Arabia | Lab-based public datasets | Experimental ML/AI study | PD | PD: 57; HC: 15 |
| Ali SM et al, (2024)^118^ | Australia | Clinical Research | Observational cross-sectional study | ET | ET: 17; HC: 18 |
| Ali SM et al, (2022)^119^ | Australia | Clinical Research | Observational cross-sectional study | ET | ET: 17; HC: 18 |
| Almeida MF et al, (2012)^28^ | Brazil | Laboratory-based Research | Observational cross-sectional study | Healthy adults | HA: 14 |
| Almeida MF et al, (2010)^36^ | Brazil | Laboratory-based Research | Observational cross-sectional study | Healthy adults | HA: 59 |
| Altmann VC et al, (2022)^56^ | Netherlands | Laboratory-based Research | Observational cross-sectional study | Other | Paralympic adult: 19 |
| Baek H et al, (2024)^58^ | US | Home/Community | Longitudinal/pre &post-intervention Study | PD & ET | ET: 37; PD: 17 |
| Bange et al, (2024)^120^ | Germany | Laboratory-based Research | Experimental ML/AI study | PD | PD: 19 |
| Banaszkiewicz K et al, (2009)^11^ | Poland | Clinical Research | Observational cross-sectional study | PD | Bradykinesia: 54; HC: 39 |
| Bernardo LS et al, (2021)^110^ | Lithuania | Lab-based public datasets | Experimental ML/AI study | PD | PD: 296; HC: 218 |
| Bui HT et al, (2017)^43^ | Canada | Laboratory-based Research | Observational cross-sectional study | Mixed movement disorders | Ataxia: 49; HC: 36 |
| Carfora D et al, (2022)^51^ | France | Clinical Research | Observational cross-sectional study | Cognitive Impairment | AD: 30; HC: 45 |
| Chandra J et al, (2021)^17^ | US | Lab-based public datasets | Observational cross-sectional study | PD | PD: 62; HC: 15 |
| Chen KH et al, (2018)^103^ | Taiwan | Clinical Research | Observational cross-sectional study | PD & ET | PD: 12; ET: 14; HC: 12 |
| Cohen et al. (2003)^121^ | US | Laboratory-based Research | Observational cross-sectional study | ET | ET: 64 |
| Creagh AP et al, (2020)^39^ | UK | Home/Community | Longitudinal/pre &post-intervention Study | MS | MS: 71; HC: 22 |
| Danna J et al, (2019)^68^ | France | Laboratory-based Research | Observational cross-sectional study | PD | PD: 20; HC: 20 |
| Darnall ND et al, (2012)^89^ | US | Clinical Research | Observational cross-sectional study | PD & ET | ET: 3; PD: 6; ET & PD: 1 |
| DelMastro HM et al, (2018)^41^ | US | Clinical Research | Observational cross-sectional study | MS | MS: 258 |
| Elble RJ et al, (1996)^7^ | US | Clinical Research | Observational cross-sectional study | ET | ET: 87 |
| Elble RJ et al, (2017)^93^ | US | Clinical Research | Observational cross-sectional study | ET | ET: 18 |
| Farhah N et al, (2024)^122^ | Saudi Arabia | Lab-based public datasets | Experimental ML/AI study | PD | PD: 51; HC: 51 |
| Feys P et al, (2007)^12^ | Belgium | Clinical Research | Observational cross-sectional study | MS | MS-Tremor: 17; MS-No-Tremor: 15; HC: 15 |
| Feys et al. (2009)^42^ | Belgium | Laboratory-based Research | Longitudinal/pre &post-intervention Study | MS | MS: 18 |
| Folador JP et al, (2021)^83^ | Brazil | Laboratory-based Research | Observational cross-sectional study | PD | PD: 20 HC: 20 |
| Fujiwara K et al, (2023)^13^ | Japan | Laboratory-based Research | Observational cross-sectional study | Cognitive Impairment | Non-MCI Youth: 10; Non-MCI Elderly:10; MCI Elderly: 10 |
| Galaz Z et al, (2022)^123^ | CZ, HU, US, CO | Lab-based public datasets | Experimental ML/AI study | PD | PD: 143; HC: 151 |
| Galli M et al, (2014)^98^ | Italy | Clinical Research | Longitudinal/pre &post-intervention Study | PD | PD: 15; HC: 15 |
| Gallicchio C et al, (2018)^124^ | Italy | Lab-based public datasets | Experimental ML/AI study | PD | PD: 61; HC: 15 |
| Gil-Martín M et al, (2019)^125^ | Spain | Lab-based public datasets | Experimental ML/AI study | PD | PD: 62; HC: 15 |
| Graça R et al, (2014)^59^ | Portugal | Home/Community | Observational cross-sectional study | PD | PD: 17; HC: 18 |
| Groznik V et al, (2015)^71^ | Slovenia | Laboratory-based Research | Observational cross-sectional study | PD & ET | PD: 46; ET: 52; Mixed tremor: 24 |
| Haubenberger D et al, (2011)^94^ | US | Clinical Research | Longitudinal/pre &post-intervention Study | ET | ET: 9 |
| Haubenberger D et al, (2013)^126^ | US | Clinical Research | Longitudinal/pre &post-intervention Study | ET | ET: 18 |
| Heintz BD et al, (2018)^72^ | US | Laboratory-based Research | Observational cross-sectional study | Healthy adults | Young: 26; Older: 24 |
| Hermle D et al, (2024)^44^ | Germany | Clinical Research | Observational cross-sectional study | Mixed movement disorders | Ataxia: 46; HC: 48 |
| Hess CW et al, (2014)^35^ | US | Clinical Research | Observational cross-sectional study | Mixed movement disorders | FT: 22; DT: 21; HC: 31 |
| Holcomb JM et al, (2023)^19^ | US | Clinical Research | Experimental ML/AI study | ET | ET: 94 |
| Hoogendam YY et al, (2015)^55^ | Netherlands | Clinical Research | Observational cross-sectional study | Other | Breast Cancer: 42; HC: 53 |
| Hoogendam YY et al, (2014)^57^ | Netherlands | Population-based Research | Observational cross-sectional study | Healthy adults | HC: 1912 |
| Hsu AW et al, (2009)^14^ | US | Clinical Research | Observational cross-sectional study | Mixed movement disorders | Niemann-Pick C: 14; HC: 14 |
| Ishii N et al, (2020)^48^ | Japan | Clinical Research | Observational cross-sectional study | ET | ET: 24; cerebellar disease: 26; HC: 41 |
| Jaichandran R et al, (2020)^127^ | India | Laboratory-based Research | Experimental ML/AI study | PD | PD: 50; HC: 50 |
| Jiang B et al, (2022)^128,129^ | US | Laboratory-based Research | Experimental ML/AI study | Healthy adults | HA: 5 |
| Jindal M et al, (2020)^129^ | India | Lab-based public datasets | Experimental ML/AI study | PD | PD: 62; HC: 15 |
| Jobbágy Á et al, (2009)^130^ | Hungary | Clinical Research | Observational cross-sectional study | PD | PD: 68 |
| Kachouri M et al, (2021)^52^ | France | Laboratory-based Research | Experimental ML/AI study | Cognitive Impairment | AD: 30; HC: 45 |
| Kalafati M et al, (2022)^131^ | Greece | Clinical Research | Observational cross-sectional study | PD | PD: 12; HC: 12 |
| Kamble M et al, (2021)^132^ | India | Lab-based public datasets | Experimental ML/AI study | PD | PD: 25; HC: 15 |
| Kan PJ et al, (2019)^90^ | Taiwan | Laboratory-based Research | Experimental ML/AI study | PD & ET | PD: 21; ET: 5; HC: 24 |
| Kim CY et al, (2019)^79^ | US | Clinical Research | Observational cross-sectional study | ET | ET: 161; HC: 80 |
| Koirala N et al, (2015)^37^ | Germany | Clinical Research | Observational cross-sectional study | Mixed movement disorders | Senile Tremor: 15; ET: 15 |
| Koppelmans V et al, (2024)^91^ | US | Clinical Research | Observational cross-sectional study | Cognitive Impairment | AD: 28; MCI: 33; HC: 53 |
| Kragelj V et al, (2014)^133^ | Slovenia | Clinical Research | Observational cross-sectional study | PD & ET | PT: 20; ET: 15; HC: 15 |
| Kraus PH et al, (2010)^101^ | Germany | Clinical Research | Observational cross-sectional study | ET | ET: ~200 |
| Kuosmanen E et al, (2019)^60^ | Finland | Home/Community | Observational cross-sectional study | PD | PD: 8; HC: 6 |
| Kuosmanen E et al, (2020)^73^ | Finland | Laboratory-based Research | Observational cross-sectional study | PD | PD: 8; HC: 6 |
| Lamba R et al, (2021)^134^ | India | Lab-based public datasets | Experimental ML/AI study | PD | PD: 62 |
| Legrand AP et al, (2017)^32^ | France | Clinical Research | Observational cross-sectional study | ET | ET: 13 |
| Li Z et al, (2022)^84^ | China | Lab-based public datasets | Observational cross-sectional study | PD | PD: 43; HC: 43 |
| Lin PC et al, (2018)^95^ | Taiwan | Clinical Research | Observational cross-sectional study | PD & ET | PD: 12; ET: 12; PD or ET: 6 |
| Liu X et al, (2005)^66^ | UK | Clinical Research | Longitudinal/pre &post-intervention Study | PD | PD: 16 |
| Longardner K et al, (2024)^30^ | US | Clinical Research | Longitudinal/pre &post-intervention Study | ET | ET: 7 |
| Longstaff MG et al, (2006)^40^ | UK | Laboratory-based Research | Observational cross-sectional study | MS | MS: 7; HC: 17 |
| Lopez-de-Ipina K et al, (2018)^9^ | Spain | Lab-based public datasets | Observational cross-sectional study | ET | ET: 21; HC: 29 |
| Lopez-de-Ipina K et al, (2021)^135^ | Spain | Lab-based public datasets | Observational cross-sectional study | ET | ET: 15; HC: 25 |
| Louis ED et al, (2012)^80^ | US | Clinical Research | Observational cross-sectional study | ET | ET: 145; HC: 34 |
| MacWilliams et al. (2021)^50^ | US | Clinical Research | Observational cross-sectional study | Galactosemia | Galactosemia: 57; HC: 80 |
| Magee R et al, (2022)^74^ | US | Laboratory-based Research | Experimental ML/AI study | Healthy adults | HA: 10 |
| Marzban et al. (2017)^136^ | Iran | Laboratory-based Research | Longitudinal/pre &post-intervention Study | PD | PD:6 |
| Memedi M et al, (2015)^61^ | Sweden | Home/Community | Longitudinal/pre &post-intervention Study | PD | PD:65 |
| Memedi M et al, (2015)^62^ | Sweden | Home/Community | Observational cross-sectional study | PD | PD: 65; HC: 10 |
| Mercaldo F et al, (2024)^85^ | Italy | Lab-based public datasets | Experimental ML/AI study | PD | 3,991 images (PD: 1,995, HC: 1,996) |
| Merchant SH et al, (2018)^81^ | US | Clinical Research | Observational cross-sectional study | ET | ET: 19 |
| Miralles F et al, (2006)^137^ | Spain | Clinical Research | Observational cross-sectional study | Mixed movement disorders | Action Tremor: 31; HC: 24 |
| Muramatsu H et al, (2024)^70^ | Japan | Laboratory-based Research | Experimental ML/AI study | Healthy adults | HA: 89 |
| Murthy GNK et al, (2023)^138^ | India | Lab-based public datasets | Experimental ML/AI study | PD | PD: 62; HC: 15 |
| Parisi L et al, (2021)^111^ | UK | Lab-based public datasets | Experimental ML/AI study | PD | PD: 105; HC: 53 |
| Pham HN et al, (2019)^139^ | Vietnam | Lab-based public datasets | Experimental ML/AI study | PD | PD: 62; HC: 15 |
| Pullman SL et al, (1998)^108^ | US | Clinical Research | Observational cross-sectional study | ET | ET: 62 |
| Purk M et al, (2023)^140^ | Germany | Clinical Research | Longitudinal/pre &post-intervention Study | PD | PD: 24; Dystonia: 26; HC: 27 |
| Radmard S et al, (2021)^106^ | US | Clinical Research | Longitudinal/pre &post-intervention Study | PD | PD: 76 |
| Rajan R et al, (2021)^104^ | India | Clinical Research | Observational cross-sectional study | Mixed movement disorders | ET: 25; DT: 25; PD: 25; HC: 25 |
| Ratliff J et al, (2018)^46^ | US | Clinical Research | Observational cross-sectional study | Mixed movement disorders | DYT1 (Tor1A): 15; DYT6 (THAP1) mutations:12; HC: 27 |
| Roth N et al, (2021)^102^ | Israel | Clinical Research | Observational cross-sectional study | ET | ET: 20; HC: 18 |
| Sadikov A et al, (2017)^63^ | Slovenia | Home/Community | Observational cross-sectional study | PD | PD: 65 |
| San Luciano M et al, (2016)^99^ | US | Clinical Research | Observational cross-sectional study | PD | PD: 138; HC: 150 |
| Sarzo-Wabi I et al, (2024)^33^ | Mexico | Lab-based public datasets | Observational cross-sectional study | PD | PD: 25; HC: 15 |
| Saunders-Pullman R et al, (2008)^100^ | US | Clinical Research | Observational cross-sectional study | PD | PD: 74 |
| Schallert W et al, (2022)^75^ | Switzerland | Clinical Research | Observational cross-sectional study | Mixed movement disorders | Patient: 29; HC: 25 |
| Schuhmayer N et al, (2017)^96^ | Austria | Clinical Research | Longitudinal/pre &post-intervention Study | ET | ET: 40 |
| Senkiv O et al, (2019)^109^ | Estonia | Laboratory-based Research | Longitudinal/pre &post-intervention Study | Other | Fatigue: 14 |
| Sisti JA et al, (2017)^76^ | US | Clinical Research | Observational cross-sectional study | Mixed movement disorders | Patient: 16; HC: 15 |
| Solé-Casals J et al, (2018)^97^ | Spain | Lab-based public datasets | Observational cross-sectional study | ET | ET: 29; HC: 21 |
| Sonnet KS et al, (2020)^141^ | US | Clinical Research | Longitudinal/pre &post-intervention Study | ET | ET: 3; HC: 2 |
| Stanley K et al, (2010)^142^ | US | Clinical Research | Observational cross-sectional study | PD | PD: 9 |
| Starita S et al, (2022)^38^ | Italy | Clinical Research | Observational cross-sectional study | Healthy adults | Elderly: 11; Young: 17 |
| Starita S et al, (2022)^31^ | Italy | Clinical Research | Observational cross-sectional study | PD | PD: 16; HC: 19 |
| Surangsrirat D et al, (2013)^143^ | Thailand | Clinical Research | Observational cross-sectional study | PD | 20 images |
| Surangsrirat D et al, (2012)^67^ | Thailand | Clinical Research | Observational cross-sectional study | PD | PD: 2 |
| Tam F et al, (2017)^144^ | Canada | Clinical Research | Longitudinal/pre &post-intervention Study | ET | ET: 12 |
| Toffoli S et al, (2021)^145^ | Italy | Clinical Research | Observational cross-sectional study | PD | PD: 30; HC: 30 |
| Toffoli S et al, (2023)^92^ | Italy | Clinical Research | Observational cross-sectional study | PD | PD: 29 |
| Ueda N et al, (2014)^45^ | Japan | Clinical Research | Observational cross-sectional study | Mixed movement disorders | Spinocerebellar degeneration: 49 |
| Ulmanová O et al, (2007)^105^ | Czech Republic | Clinical Research | Observational cross-sectional study | ET | ET: 14; HC: 14 |
| Vaidya OV et al, (2024)^86^ | India | Lab-based public datasets | Observational cross-sectional study | PD | 240 images |
| Valla E et al, (2022)^146^ | Estonia | Clinical Research | Observational cross-sectional study | PD | PD: 24; HC: 34 |
| Valla E et al, (2023)^54^ | Estonia | Clinical Research | Observational cross-sectional study | Other | Fatigue: 41 |
| Virmani, T (2022)^65^ | US | Telemedicine/Home | Observational cross-sectional study | PD | PD:50 |
| Wang B et al, (2007)^147^ | China | Clinical Research | Observational cross-sectional study | PD | PD: 6; HC: 2 |
| Wang H et al, (2008)^148^ | US | Clinical Research | Observational cross-sectional study | Mixed movement disorders | PD: 10; ET: 10; DY: 10; HC: 10 |
| Wang M et al, (2012)^112^ | China | Clinical Research | Observational cross-sectional study | PD | PD: 12; HC: 3 |
| Wang Y et al, (2023)^18^ | China | Clinical Research | Observational cross-sectional study | ET | ET: 50; HC: 40 |
| Westin J et al, (2010)^64^ | Sweden | Home/Community | Longitudinal/pre &post-intervention Study | PD | PD: 60 |
| Wille A et al, (2013)^149^ | Germany | Clinical Research | Observational cross-sectional study | Mixed movement disorders | 109 drawings |
| Wrobel K et al, (2022)^87^ | Poland | Lab-based public datasets | Observational cross-sectional study | PD | PD: 62; HC: 15 |
| Zham P et al, (2018)^88^ | Australia | Clinical Research | Observational cross-sectional study | PD | PD: 31; HC: 31 |
| Zhao et al. (2024)^49^ | China | Laboratory-based Research | Observational cross-sectional study | Cerebral small vessel disease (CSVD) | Severe CSVD: 16; Non-severe: 12; HC: 32 |
