# Supplementary material for: Clinical Applications and Measurement Properties of the Digitized Archimedes Spiral Drawing Test: A Scoping Review
Source: Mov Disord Clin Pract. 2025 Aug 7;12(11):1742–55. doi: 10.1002/mdc3.70278 (PMC12625189; doi:10.1002/mdc3.70278)
Supplement: Supplementary file 5 — Table S5. Device used in the studies of spiral drawing test. [file MDC3-12-1742-s001.docx]

## Table S5. Device Used in the Studies of Spiral Drawing Test

| **Device Category** | **Device Type** | **Count (n)** | **Percentage (%)** |
| --- | --- | --- | --- |
| **Digital graphic tablet** | Wacom Graphic Tablet | 41 | 34.2 |
|  | Other | 16 | 13.3 |
|  | iPad | 6 | 5.0 |
|  | Unspecified | 17 | 14.2 |
| **Digitized paper version** | Paper scanned | 11 | 9.2 |
|  | Photo of paper-based test | 1 | 0.8 |
| **Smartphone** | Smartphone | 8 | 6.7 |
| **Digital Pen** | Digital Pen | 6 | 5.0 |
| **Wearable sensors** | Wacom Graphic Tablet | 1 | 0.8 |
|  | Wearable system with VR controller | 1 | 0.8 |
|  | Other | 1 | 0.8 |
| **Unknown** | Unspecified | 11 | 9.2 |
| **Total** |  | 120 | 100 |
