# Supplementary material for: Clinical Applications and Measurement Properties of the Digitized Archimedes Spiral Drawing Test: A Scoping Review
Source: Mov Disord Clin Pract. 2025 Aug 7;12(11):1742–55. doi: 10.1002/mdc3.70278 (PMC12625189; doi:10.1002/mdc3.70278)
Supplement: Supplementary file 6 — Table S6. Drawing task, device, and analysis method for all included studies. [file MDC3-12-1742-s007.docx]

## Table S6. Drawing Task, Device, and Analysis Method for All Included Studies

| **First Author (Year)** | **Device used** | **Drawing Task** | **Instruction** | **Hand Analyzed** | **No of drawings collected per participant** | **Spiral Metrics** | **Analysis Method** |
| --- | --- | --- | --- | --- | --- | --- | --- |
| Aghanavesi S et al, (2017)^77^ | Smartphone with stylus | Trace a spiral template | as fast (within 10 s) and accurately as possible | Dominant hand | 15 | Temporal Irregularity Score (TIS) | Machine Learning & Deep Learning |
| Aghanavesi S et al, (2017)^78^ | Smartphone with stylus | Trace a spiral template | as fast (within 10 s) and accurately as possible | Dominant hand | 15 | 37 features (e.g., speed, radial velocity, Approximate Entropy). | Hypothesis-Driven Statistical Analysis |
| Al-Yousef N et al, (2020)^82^ | Digital tablet | Dynamic Spiral Test | Unknown | Unknown | Unknown | ML features | Machine Learning & Deep Learning |
| Ali SM et al, (2024)^118^ | Wacom Intuos Pro | Did not report | natural speed | Unknown | Unknown | Power spectral density (PSD) ratio, Total PSD, Scalar magnitude, Total task time | Machine Learning |
| Ali SM et al, (2022)^119^ | Wacom Intuos Pro | Draw between guidelines | natural speed | Dominant hand | Unknown | Power spectral density (PSD) ratio, the vector magnitude of acceleration data across all three axes (x, y, z) | Hypothesis-Driven Statistical Analysis |
| Almeida MF et al, (2012)^28^ | Trust, model TB-4200 | Trace a spiral template | natural speed | Dominant hand | 6 | Linear Discriminant Analysis (LDA)-values | Hypothesis-Driven Statistical Analysis |
| Almeida MF et al, (2010)^36^ | Trust, model TB-4200 | Trace a spiral template | natural speed | Dominant hand | 6 | Frequency, standard deviation, approximate entropy, and LDA-value | Hypothesis-Driven Statistical Analysis |
| Altmann VC et al, (2022)^56^ | Wacom Cintiq 16 | Draw between guidelines | Unknown | Dominant and nondominant hand | 6 | Movement time | Hypothesis-Driven Statistical Analysis |
| Baek H et al, (2024)^58^ | Paper scanned | Draw between guidelines | Unknown | Unknown | 1 | Line length, curvature, and crossing points | Hypothesis-Driven Statistical Analysis |
| Banaszkiewicz K et al, (2009)^11^ | Wacom GD-1212R | Trace a spiral template | natural speed | Dominant and nondominant hand | 6 | Spiral Drawing time | Hypothesis-Driven Statistical Analysis |
| Bange et al, (2024)^120^ | Wacom Intuos Pro - Creative Pen Tablet | Free hand & Trace template | natural speed | Dominant hand | Up to 12 | Average velocity and spatial accuracy | Hypothesis-Driven Statistical Analysis |
| Bernardo LS et al, (2021)^110^ | Digital and paper-based | Did not report | Unknown | Unknown | Unknown | 512 deep features extracted by CNN, Kinematic features, Time and frequency domain features, and preprocessing features | Machine Learning & Deep Learning |
| Bui HT et al, (2017)^43^ | Touch-screen computer & Index Finger | Freehand drawing | natural speed | Dominant hand | 1 | Mean error, maximum error, Spatial Displacement (Radial Error), Smoothness of Movement (Frequency Analysis), Execution time | Hypothesis-Driven Statistical Analysis |
| Carfora D et al, (2022)^51^ | Wacom Intuos Pro | Did not report | Unknown | Unknown | 1 | Pen pressure, altitude, velocity, and acceleration | Machine Learning & Deep Learning |
| Chandra J et al, (2021)^17^ | Wacom Cintiq 12WX with digital pen | Dynamic Spiral Test | Unknown | Unknown | 2 | Velocity, acceleration, jerk, curvature, radius vs theta regression | Machine Learning |
| Chen KH et al, (2018)^103^ | Wacom Cintiq 13HD | Dynamic Spiral Test | Unknown | Severe hand or dominant hand | 3 | Absolute velocity, SD of velocity, tracking performance ratio | Hypothesis-Driven Statistical Analysis |
| Cohen et al. (2003)^121^ | Digital tablet | Did not report | Unknown | Dominant and nondominant hand | 20 | spiral width and height, spiral pen distance traveled | Hypothesis-Driven Statistical Analysis |
| Creagh AP et al, (2020)^39^ | Smartphone & finger | Freehand drawing | as fast (within 30 s) and as accurate as possible | Dominant and nondominant hand | 1 | Drawing velocity, angular and radial velocities, drawing error (using Hausdorff distance), and image entropy | Hypothesis-Driven Statistical Analysis |
| Danna J et al, (2019)^68^ | Wacom Intuos 3 and ink pen | Trace a spiral template | natural speed & as fast as possible | Dominant and nondominant hand | 6 | Pen altitude, velocity peaks, and pen azimuth variations | Hypothesis-Driven Statistical Analysis |
| Darnall ND et al, (2012)^89^ | Digital paper and pen (Adapx’s Capturx) | Trace a spiral template | Unknown | Unknown | 2 | Root mean squared [RMS] angular velocities) and digital pen data to assess tremor severity | Machine Learning |
| DelMastro HM et al, (2018)^41^ | Wacom Intuos Pro and digital pen | Draw between guidelines | natural speed | Dominant hand | 2 | Number of segments in the drawing (SEGRT), SD of Radial Velocity (VSD-R); SD of Tangential Velocity (VSD-T); SD of Overall Velocity (VSD-O); Mean Drawing Velocity (MNV-O), and Mean Pen Pressure Acceleration (MNA-P) | Hypothesis-Driven Statistical Analysis |
| Elble RJ et al, (1996)^7^ | Digital tablet | Mixed method | natural speed | Unknown | Unknown | Tremor frequency, amplitude, pen lift, pen pressure, velocity | Hypothesis-Driven Statistical Analysis |
| Elble RJ et al, (2017)^93^ | Digital tablet | Trace a spiral template | Unknown | Dominant and nondominant hand | 4 | Peak-to-peak tremor displacement, log-transformed tremor amplitude | Hypothesis-Driven Statistical Analysis |
| Farhah N et al, (2024)^122^ | NA | Did not report | Unknown | Unknown | Unknown | Speed, pressure, and movement irregularities, and digital image properties | AI/Deep Learning |
| Feys P et al, (2007)^12^ | Easypen G6 & digital pen | Draw between guidelines | natural speed while accuracy was emphasized | Dominant and nondominant hand | 4 | SD of velocity, overall drawing velocity (SD-DV) | Hypothesis-Driven Statistical Analysis |
| Feys et al. (2009)^42^ | Easypen G6 | Trace a spiral template | natural speed | Dominant hand | Unknown | SD of overall drawing velocity, radial and tangential direction | Hypothesis-Driven Statistical Analysis |
| Folador JP et al, (2021)^83^ | Paper scanned | Freehand drawing | Unknown | Dominant hand | 6 | HOG features capture gradient information related to the shapes of the drawn patterns | Machine Learning & Deep Learning |
| Fujiwara K et al, (2023)^13^ | Surface Pro 4 tablet and Stylus pen | Mixed method | complete the drawing within 10 s | Dominant hand | 1 | Deviation from the centerline, Direction of Deviation (ratio of inward displacement to outward displacement) | Hypothesis-Driven Statistical Analysis |
| Galaz Z et al, (2022)^123^ | Wacom Cintiq 13HD | Did not report | Unknown | Unknown | Unknown | CNN-learned features and handcrafted features (such as kinematic, temporal-spatial, and velocity-based metrics) | AI/Deep Learning |
| Galli M et al, (2014)^98^ | Optoelectronic system with six cameras (SMART BTS) | Freehand drawing | natural speed | Unknown | Unknown | Spiral Dimension (D), Velocity Profiles, Angular Coefficient of Velocity Regression | Hypothesis-Driven Statistical Analysis |
| Gallicchio C et al, (2018)^124^ | Digital tablet | Did not report | Unknown | Unknown | Unknown | Pen position, pressure, and grip angle | AI/Deep Learning |
| Gil-Martín M et al, (2019)^125^ | Wacom Cintiq 12WX with digital pen | Dynamic Spiral Test | Unknown | Unknown | Unknown | Fast Fourier Transform (FFT) to create frequency spectra as input for the CNN | AI/Deep Learning |
| Graça R et al, (2014)^59^ | Smartphone with finger and stylus | Trace a spiral template | Unknown | Unknown | Unknown | Spiral Average Error, Spiral Cross, Spiral Pressure Ratio, Spiral Side Ratio | Hypothesis-Driven Statistical Analysis |
| Groznik V et al, (2015)^71^ | Mobile device with fingers | Freehand drawing | Unknown | Unknown | Unknown | Radial speed, frequency analysis, and symmetry | Hypothesis-Driven Statistical Analysis |
| Haubenberger D et al, (2011)^94^ | Wacom Intuos 3 | Draw between guidelines | natural speed | Dominant hand | 1 | Velocity Tremor Peak Amplitude, Spectral Tremor Peak Frequency, AUC around the spectral peak | Hypothesis-Driven Statistical Analysis |
| Haubenberger D et al, (2013)^126^ | NA | Did not report | Unknown | Unknown | Unknown | Tremor power over time | Hypothesis-Driven Statistical Analysis |
| Heintz BD et al, (2018)^72^ | iPad mini using both a stylus and finger | Trace a spiral template | natural speed while accuracy was emphasized | Unknown | 3 | Root Mean Square (RMS) Error, Downward Pressing Force, completion time | Hypothesis-Driven Statistical Analysis |
| Hermle D et al, (2024)^44^ | Digitizer pen (Polhemus FASTRAK) | Trace a spiral template | natural speed while accuracy was emphasized | Dominant hand | 2 | Speed, smoothness, variability, efficiency, and endpoint precision | Hypothesis-Driven Statistical Analysis |
| Hess CW et al, (2014)^35^ | Wacom Intuos 4 with stylus | Freehand drawing | Unknown | Dominant and nondominant hand | 20 | Spiral severity score and inter-spiral tightness variability | Hypothesis-Driven Statistical Analysis |
| Holcomb JM et al, (2023)^19^ | Digital tablet | Freehand drawing | Unknown | Dominant and nondominant hand | 4 | ML/DL metrics | AI/Deep Learning |
| Hoogendam YY et al, (2015)^55^ | Wacom Graphire & wireless pen | Trace a spiral template | as accurately and as fast as possible | Dominant hand | 1 | Movement time, drawing length, average speed, speed variability, deviation from the template, and return movements | Hypothesis-Driven Statistical Analysis |
| Hoogendam YY et al, (2014)^57^ | Wacom Graphire & wireless pen | Trace a spiral template | as accurately and as fast as possible | Dominant hand | 1 | Movement time, length of drawing, average speed, speed variability, deviation from template, and number of crossings. | Hypothesis-Driven Statistical Analysis |
| Hsu AW et al, (2009)^14^ | Wacom Intuos 2 | Freehand drawing | Unknown | Dominant and nondominant hand | 20 | Drawing speed, acceleration, loop width variability, tremor frequency, and pressure profiles | Hypothesis-Driven Statistical Analysis |
| Ishii N et al, (2020)^48^ | Photo of paper-based test | Trace a spiral template | Unknown | Unknown | 1-3 | Percentage of spiral length and total deviation area | AI/Deep Learning |
| Jaichandran R et al, (2020)^127^ | NA | Did not report | Unknown | Unknown | Unknown | X, Y, and Z coordinates, timestamp, pressure, and grip angle values, PCA | Machine Learning |
| Jiang B et al, (2022)^128,129^ | NA | Trace a spiral template | Unknown | Unknown | Unknown | Tremor power, Dominant frequency, completion time, outside area, task-specific variations | Hypothesis-Driven Statistical Analysis |
| Jindal M et al, (2020)^129^ | Digital tablet | Dynamic Spiral Test | Unknown | Unknown | Unknown | X, Y, and Z coordinates, pressure, grip angle, frequency domain features | AI/Deep Learning |
| Jobbágy Á et al, (2009)^130^ | Digital tablet | Did not report | Unknown | Unknown | Unknown | Deviations from a reference spiral, high pass filtered kinetic tremor signals, and trajectory tracking errors | Hypothesis-Driven Statistical Analysis |
| Kachouri M et al, (2021)^52^ | Wacom Intuos Pro | Freehand drawing | Unknown | Unknown | 1 | Pen pressure, altitude, velocity, PCA | Machine Learning |
| Kalafati M et al, (2022)^131^ | Android tablet and Sonar pen (smart stylus) | Dynamic Spiral Test | Unknown | Unknown | Unknown | Spiral deviation, total time, and pen pressure | Hypothesis-Driven Statistical Analysis |
| Kamble M et al, (2021)^132^ | Wacom Cintiq 12WX with digital pen | Dynamic Spiral Test | Unknown | Unknown | Unknown | Pressure, grip angle, radial velocity, stroke speed, number of strokes, in-air versus on-surface time, acceleration, and jerk | Machine Learning |
| Kan PJ et al, (2019)^90^ | Apple iPad Pro with Apple Pencil | Freehand drawing | natural speed | Dominant hand | 2 | Deviation, Accumulation Angle | Machine Learning |
| Kim CY et al, (2019)^79^ | Wacom Intuos 4 | Freehand drawing | Unknown | Dominant and nondominant hand | 20 | Degree of Severity (DoS), Tremor Amplitude (Ampl) | Hypothesis-Driven Statistical Analysis |
| Koirala N et al, (2015)^37^ | Wacom Intuos 4 | Mixed method | Unknown | Dominant and nondominant hand | 8 | DoS, Peak Frequency, Peak Amplitude, spiral width, height, and axis | Hypothesis-Driven Statistical Analysis |
| Koppelmans V et al, (2024)^91^ | Wacom Intuos | Trace a spiral template | as accurately and as fast as possible | Dominant hand | 1 | Movement time, length of drawing, average speed, speed variability, template deviation, and return movements | Machine Learning |
| Kragelj V et al, (2014)^133^ | Digital tablet | Freehand drawing | Unknown | Unknown | Unknown | Spiral image, tremor spectrum evaluation, the wavelet transforms scalogram | Hypothesis-Driven Statistical Analysis |
| Kraus PH et al, (2010)^101^ | Paper scanned | Trace a spiral template | Unknown | Unknown | Unknown | Tremor amplitude | Hypothesis-Driven Statistical Analysis |
| Kuosmanen E et al, (2019)^60^ | Android smartphone-based with finger | Freehand drawing | Unknown | Unknown | Unknown | Av. error in spiral, drawing speed | Hypothesis-Driven Statistical Analysis |
| Kuosmanen E et al, (2020)^73^ | Android smartphone-based with finger | Trace a spiral template | Unknown | Unknown | Unknown | Drawing speed and total time, drawing accuracy (error), Crossing rate (as percentage), Radial and angular velocity, Drawing sampling rate and gaps | Hypothesis-Driven Statistical Analysis |
| Lamba R et al, (2021)^134^ | Digital tablet | Dynamic Spiral Test | Unknown | Unknown | Unknown | Number of strokes, speed, velocity, acceleration, jerk, horizontal and vertical movement characteristics, changes in velocity and acceleration (NCV and NCA), in-air time, and on-surface time | Machine Learning |
| Legrand AP et al, (2017)^32^ | Wacom Bamboo Fun Medium | Mixed method | natural speed | Dominant hand | 4 | V score, EMD scores, URS score, URD scores | Hypothesis-Driven Statistical Analysis |
| Li Z et al, (2022)^84^ | Paper scanned | Freehand drawing | Unknown | Unknown | 3-5 | AI- Deep learning image features | AI/Deep Learning |
| Lin PC et al, (2018)^95^ | Wacom Cintiq 13HD | Mixed method | Unknown | Dominant and nondominant hand | 6 | The means of radial difference per radian (\|dr/dθ\|), the means of radial difference per second (\|dr/dt\|), and the AUC of the frequency spectrum of the velocity | Hypothesis-Driven Statistical Analysis |
| Liu X et al, (2005)^66^ | Easypen G6 & digital pen | Trace a spiral template | natural speed | Dominant and nondominant hand | Unknown | SD of drawing velocity (SD-DV) | Hypothesis-Driven Statistical Analysis |
| Longardner K et al, (2024)^30^ | ThinkPad X60 tablet PC and a magnetic pen | Trace a spiral template | natural speed | Dominant and nondominant hand | 4 | Mean and maximum tremor amplitude | Hypothesis-Driven Statistical Analysis |
| Longstaff MG et al, (2006)^40^ | Wacom 1212-R | Trace a spiral template | natural speed while accuracy was emphasized | Dominant hand | Unknown | Axial pen pressure, Tangential velocity, Trajectory variability, Scaling Ratio for Spiral Radius | Hypothesis-Driven Statistical Analysis |
| Lopez-de-Ipina K et al, (2018)^9^ | Wacom Intuos 4 | Freehand drawing | Unknown | Dominant and nondominant hand | Unknown | Classic linear features (static and dynamics) and non-linear features (fractal dimension and entropy) | Machine Learning |
| Lopez-de-Ipina K et al, (2021)^135^ | Wacom Intuos 4 | Freehand drawing | Unknown | Unknown | Unknown | Spatial coordinates (x, y), pressure, azimuth and altitude angles of the pen, and their variations (delta and delta-delta) | Hypothesis-Driven Statistical Analysis |
| Louis ED et al, (2012)^80^ | Digital tablet | Freehand drawing | Unknown | Dominant and nondominant hand | 20 | Spiral width variability index (SWVI) | Hypothesis-Driven Statistical Analysis |
| MacWilliams et al. (2021)^50^ | Surface Pro | Draw between guidelines | natural speed | Dominant and nondominant hand | 4 | Deviation from an idealized spiral (RMS), velocity and frequency in the range of 4-8 Hz (FFT) | Hypothesis-Driven Statistical Analysis |
| Magee R et al, (2022)^74^ | Web-based application | Mixed method | Unknown | Dominant hand | 30 | Mean intra-cluster distance | Hypothesis-Driven Statistical Analysis |
| Marzban et al. (2017)^136^ | Wacom Intuos 2 | Did not report | Unknown | Dominant hand | Unknown | Overall time, speed, acceleration, Approximate Entropy (ApEn), PC1, ratio of power in 0.1-5Hz to power in 5-10 Hz | Hypothesis-Driven Statistical Analysis |
| Memedi M et al, (2015)^61^ | Qtek 2020i Pocket PC device | Trace a spiral template | Unknown | Dominant hand | 3 | Mean spiral score (MSS), Overall test score (OTS) | Hypothesis-Driven Statistical Analysis |
| Memedi M et al, (2015)^62^ | Touch screen telemetry device with a stylus | Trace a spiral template | as accurately and as fast as possible | Dominant hand | 3 | Spatiotemporal features (e.g. Mean Drawing Speed, Approximate Entropy, Root Mean Square Error (RMSE), 28 extracted features resulted in 4 PCs | Machine Learning |
| Mercaldo F et al, (2024)^85^ | NA | Did not report | Unknown | Unknown | Unknown | AI- Deep learning image features | AI/Deep Learning |
| Merchant SH et al, (2018)^81^ | Wacom Intuos 2–4 | Freehand drawing | Unknown | Dominant and nondominant hand | 20 | SWVI, Degree of Severity (DoS) | Hypothesis-Driven Statistical Analysis |
| Miralles F et al, (2006)^137^ | Paper scanned | Trace a spiral template | natural speed | Dominant hand | 1 | Spatial cross-correlation coefficient, radial error (mean and sd), FFT analysis by sectors | Hypothesis-Driven Statistical Analysis |
| Muramatsu H et al, (2024)^70^ | Wacom DTK-2200 | Trace a spiral template | complete the movement in exactly 6 s and prioritize temporal accuracy over spatial reproduction | Dominant hand | 174 | Spatial error (distance between the stylus and the desired spiral path) and temporal error (timing differences along the spiral trajectory), Trajectory and Velocity Profiles, Force metrics, performance improvement | Hypothesis-Driven Statistical Analysis |
| Murthy GNK et al, (2023)^138^ | Wacom Cintiq 12WX with digital pen | Dynamic Spiral Test | Unknown | Unknown | Unknown | Spatial data (X, Y, and Z coordinates), temporal variations, weight (pressure applied), and grip angle | AI/Deep Learning |
| Parisi L et al, (2021)^111^ | NA | Did not report | Unknown | Unknown | Unknown | AI Deep learning 784 features per image | AI/Deep Learning |
| Pham HN et al, (2019)^139^ | NA | Dynamic Spiral Test | Unknown | Unknown | Unknown | X, Y, and Z positions of the pen (representing horizontal, vertical, and perpendicular movements, respectively), pressure applied on the screen, grip angle, system time for each sample | Machine Learning |
| Pullman SL et al, (1998)^108^ | Digital tablet (Kurta) | Freehand drawing | Unknown | Dominant and nondominant hand | 20 | First-order smoothness, Second-order smoothness, Tightness, Zero crossing rate, Second-order zero-crossing rate, peak frequency and power in the X, Y, pressure axes, DoS | Hypothesis-Driven Statistical Analysis |
| Purk M et al, (2023)^140^ | Apple iPad Pro with Apple Pencil | Freehand drawing | natural speed | Dominant and nondominant hand | 4 | Motor symptoms, non‑motor symptoms features, Precision features, Force features, Time-related features, | Machine Learning |
| Radmard S et al, (2021)^106^ | Wacom Intuos 4 with ink pen | Freehand drawing | Unknown | Dominant and nondominant hand | 20 | Severity, Smoothness, Tremor, Variability, and Tightness | Hypothesis-Driven Statistical Analysis |
| Rajan R et al, (2021)^104^ | Paper scanned | Freehand drawing | Unknown | Dominant hand | 3 | Total deviation from ideal spiral (mean deviation, SD of Tremor Variability | Hypothesis-Driven Statistical Analysis |
| Ratliff J et al, (2018)^46^ | Digital tablet | Freehand drawing | Unknown | Dominant and nondominant hand | 20 | DoS, first- and second order drawing smoothness (1st OrderSm, 2nd OrderSm), residual of the radius versus the angle, theta (ResThetaR), first- and second-order zero-crossing (1st ZC, 2nd ZC), SWVI, mean Pressure, the pressure frequency (PFreq), and power of pressure frequency (PFreqP) | Hypothesis-Driven Statistical Analysis |
| Roth N et al, (2021)^102^ | Wacom Intuos 2 with an ink pen | Draw between guidelines | Unknown | Unknown | Unknown | Total task duration, total drawing length, normalized total length of the drawn curve, mean drawing velocity etc 10 measures | Hypothesis-Driven Statistical Analysis |
| Sadikov A et al, (2017)^63^ | Digital device (unspecified) | Trace a spiral template | as accurately and as fast as possible | Dominant hand | 3 | Radial speed variability, Tangential speed variability, Absolute speed variability, Level of curvature/smoothness of the spiral.. Etc. 10 features | Machine Learning |
| San Luciano M et al, (2016)^99^ | Wacom Intuos 4 with ink pen | Freehand drawing | Unknown | Dominant and nondominant hand | 20 | Overall DoS spiral execution, measures of shape and kinematics based on 2ndSm and 1stZC, tightness (T), mean drawing speed (mSp) and a SWVI | Hypothesis-Driven Statistical Analysis |
| Sarzo-Wabi I et al, (2024)^33^ | Paper scanned | Did not report | Unknown | Dominant hand | Unknown | 5 groups of features: Pressure, Texture, Morphologic, Kinematic, Frequency | Machine Learning |
| Saunders-Pullman R et al, (2008)^100^ | Wacom Intuos 2 with an ink pen | Freehand drawing | Unknown | Dominant and nondominant hand | 20 | 1st ZC, 2nd OrderSm, mSp, DoS | Hypothesis-Driven Statistical Analysis |
| Schallert W et al, (2022)^75^ | iPad Air tablet with a web-based app | Trace a spiral template | as accurately as possible in 20 second | Dominant and nondominant hand | 5 | Speed, accuracy and path length | Hypothesis-Driven Statistical Analysis |
| Schuhmayer N et al, (2017)^96^ | Digital tablet | Did not report | Unknown | Dominant and nondominant hand | 20 | Tremor amplitude measures and frequencies | Hypothesis-Driven Statistical Analysis |
| Senkiv O et al, (2019)^109^ | Digital tablet | Trace a spiral template | Unknown | Unknown | Unknown | Fisher’s score with temporal features and non-temporal features | Machine Learning & Deep Learning |
| Sisti JA et al, (2017)^76^ | Wacom Tablet with stylus, iPad with index finger | Freehand drawing | Unknown | Dominant and nondominant hand | 20 | Degree of Severity (DoS) | Hypothesis-Driven Statistical Analysis |
| Solé-Casals J et al, (2018)^97^ | Wacom Intuos 4 | Did not report | Unknown | Dominant and nondominant hand | Unknown | Electrophysiological test features | Machine Learning |
| Sonnet KS et al, (2020)^141^ | NA | Draw between guidelines | Unknown | Unknown | Unknown | FFT and Discrete Wavelet Transform (DWT) tremor features | Machine Learning |
| Stanley K et al, (2010)^142^ | Wacom Intuos 2 with an ink pen | Freehand drawing | Unknown | Dominant and nondominant hand | 20 | Graphonometric methods assessing curvature, drawing speed and other kinematic measures | Hypothesis-Driven Statistical Analysis |
| Starita S et al, (2022)^38^ | Wacom Intuos 3 with ink pen | Trace a spiral template | natural speed while accuracy was emphasized | Unknown | Unknown | Kinematic parameters extracted at whole track and at stroke level | Hypothesis-Driven Statistical Analysis |
| Starita S et al, (2022)^31^ | Wacom Intuos 3 with ink pen | Trace a spiral template | natural speed while accuracy was emphasized | Unknown | 1 | Mean pressure (P), mean duration of strokes (Ds), mean curvilinear velocity of strokes (Vcs), stroke number normalized by total track length (Ns/L), and normalized jerk (JN) | Hypothesis-Driven Statistical Analysis |
| Surangsrirat D et al, (2013)^143^ | Android mobile device with stylus | Trace a spiral template | natural speed | Dominant and nondominant hand | 4 | FFT tremor metrics | Hypothesis-Driven Statistical Analysis |
| Surangsrirat D et al, (2012)^67^ | Android mobile device with stylus | Trace a spiral template | natural speed | Unknown | 2 | Average time, SD, radial error, SD, Crossing rate, Hemi-spiral pressure ratio | Hypothesis-Driven Statistical Analysis |
| Tam F et al, (2017)^144^ | MRI-compatible touch tablet and stylus | Trace a spiral template | natural speed | Dominant and nondominant hand | 4 | FFT tremor metrics | Hypothesis-Driven Statistical Analysis |
| Toffoli S et al, (2021)^145^ | Smart ink pen | Trace a spiral template | no constraints were imposed on the accuracy and on the speed of execution | Dominant and nondominant hand | Unknown | 10 time domain indicators and 3 frequency domain indicators | Hypothesis-Driven Statistical Analysis |
| Toffoli S et al, (2023)^92^ | Smart ink pen | Trace a spiral template | Unknown | Dominant and nondominant hand | 4 | 45 relevant indicators, divided into 7 domains | Machine Learning |
| Ueda N et al, (2014)^45^ | Paper scanned | Trace a spiral template | natural speed while accuracy was emphasized | Dominant hand | Unknown | The gap between the template spiral and the drawn spiral (GA) | Hypothesis-Driven Statistical Analysis |
| Ulmanová O et al, (2007)^105^ | XGT (Kurta Corporation, Phoenix, AZ, USA) and a non-inking pen | Draw between guidelines | unknown | Dominant and nondominant hand | 1 | Tremor magnitude | Hypothesis-Driven Statistical Analysis |
| Vaidya OV et al, (2024)^86^ | NA | Did not report | Unknown | Unknown | Unknown | AI- Deep learning image recognition | AI/Deep Learning |
| Valla E et al, (2022)^146^ | Apple iPad Pro with Apple Pencil | Did not report | Unknown | Unknown | Unknown | 202 features were extracted from the raw signals | Machine Learning |
| Valla E et al, (2023)^54^ | Smartphones app on Android OS device | Draw between guidelines | Unknown | Unknown | Unknown | 60 distinct features such as kinematic, angular, aim-based, and tremor-related measures (e.g. distance, acceleration, 𝜙_angle_mass, 𝑥_jerk_mass, crackle_mass) | Machine Learning |
| Virmani, T (2022)^65^ | Paper scanned | Did not report | Unknown | Dominant and nondominant hand | 2 | spiral width and height, spiral pen distance traveled, spiral area | Hypothesis-Driven Statistical Analysis |
| Wang B et al, (2007)^147^ | Digital tablet (GRAPHTEC KD4300) and a digitized pen | Freehand drawing | Unknown | Unknown | Unknown | Max Radius, Slope, Mean and SD of Sx and Sy, Number of turns | Hypothesis-Driven Statistical Analysis |
| Wang H et al, (2008)^148^ | Digital tablet | Freehand drawing | Unknown | Unknown | 10 | Optimal spiral center detection in different frequency domains | Hypothesis-Driven Statistical Analysis |
| Wang M et al, (2012)^112^ | Digital tablet (GRAPHTEC KD4300) and a digitized pen | Freehand drawing | Unknown | Unknown | Unknown | Maximum radius Rmax, Slope θ, Mean and standard deviation of the value of the x-, y-coordinate, Number of turns n | Hypothesis-Driven Statistical Analysis |
| Wang Y et al, (2023)^18^ | Paper scanned | Freehand drawing | Unknown | Unknown | 1-2 | A variety of features generated by CNN model | AI/Deep Learning |
| Westin J et al, (2010)^64^ | Qtek 2020i Pocket PC device | Trace a spiral template | as accurately as possible | Dominant hand | 1 | Wavelet spiral test score (WSTS), SD of frequency-filtered drawing velocity (SDDV) | Hypothesis-Driven Statistical Analysis |
| Wille A et al, (2013)^149^ | Paper scanned | Did not report | Unknown | Unknown | Unknown | Relative line orientations | Hypothesis-Driven Statistical Analysis |
| Wrobel K et al, (2022)^87^ | Wacom Cintiq 12WX with digital pen | Did not report | Unknown | Unknown | Unknown | 14 spatial temporal and pressure features extracted by ML-classifier | Machine Learning |
| Zham P et al, (2018)^88^ | Wacom Intuos Pro Large with ink pen | Trace a spiral template | Unknown | Unknown | Unknown | 14 spatial temporal (speed, position, pressure) and pressure features extracted by ML-classifier | Machine Learning |
| Zhao et al. (2024)^49^ | Wacom Cintiq DTK1661 | Did not report | Unknown | Dominant hand | 3 | Average Normalized Jerk (ANJ) | Hypothesis-Driven Statistical Analysis |
