# Supplementary material for: Clinical Applications and Measurement Properties of the Digitized Archimedes Spiral Drawing Test: A Scoping Review
Source: Mov Disord Clin Pract. 2025 Aug 7;12(11):1742–55. doi: 10.1002/mdc3.70278 (PMC12625189; doi:10.1002/mdc3.70278)
Supplement: Supplementary file 7 — Table S7. COSMIN (Consensus‐Based Standards for the Selection of Health Measurement Instruments) measurement properties and application to DAST (digitized Archimedes spiral drawing test). [file MDC3-12-1742-s006.docx]

## Table S7. COSMIN Measurement Properties and Application to DAST

| **Measurement Property Category** | **Measurement Property** | **COSMIN Definition** | **Relation to DAST** | **Application in ML/DL** |
| --- | --- | --- | --- | --- |
| **Reliability** | Measurement Consistency (includes intra-rater, inter-rater, and test-retest reliability) | The degree to which repeated measurements under similar conditions yield consistent results, whether across time, raters, or systems. | Applies to DAST in multiple ways: (1) test-retest of repeated drawings by the same person, (2) comparisons between human and algorithm ratings, or (3) comparisons across different models or feature sets. | Relevant for evaluating consistency of predictions across multiple model runs, raters, or software versions. Examples include comparing CNN outputs with human experts, or repeated spiral input tests. |
|  | Measurement error | The systematic and random error of a measurement that is not attributed to true score differences. | Applies to DAST to assess errors in digital metrics, such as variations in recorded spiral dimensions. | Assesses the noise or error in model predictions caused by data inconsistencies or model design. |
| **Validity** | Content validity | The extent to which the content of a measurement instrument is an adequate reflection of the construct to be measured. | Applies to ensure that the test adequately reflects fine motor function constructs, such as precision and tremor. | Ensures that the features and inputs used by the model comprehensively represent the construct being modeled. |
|  | Construct validity (Convergent validity) | The degree to which a measure correlates with other measures of the same construct. | Applies to DAST by correlating its metrics with other fine motor tests (e.g., pegboard tasks, handwriting assessments). | Evaluates the correlation between model predictions and other validated tools measuring the same construct. |
|  | Construct validity (Discriminant or known-group validity) | The ability of a measure to distinguish between predefined groups (known-group validity) or detect meaningful differences between distinct constructs (discriminant validity). | Applies to DAST when identifying differences between clinical and non-clinical populations or groups with varying motor function. | Relevant for ML/DL models that classify populations or features based on distinct patterns of motor function or disease states. |
|  | Criterion validity (Concurrent validity) | The degree to which a measure correlates with a criterion measure taken at the same time. | Applies to DAST by comparing its metrics with gold-standard measures of fine motor function (e.g., traditional spiral drawing tests). | Assesses whether the ML/DL model aligns with outputs from gold-standard tools or human experts. |
|  | Criterion validity (Predictive validity) | The degree to which a measure predicts future outcomes or behaviors. | Applies to DAST in predicting future clinical outcomes (e.g., motor decline or disease progression). | Tests whether the model can predict future outcomes or trends based on current data. |
| **Responsiveness** | Responsiveness | The ability of a measure to detect change over time. | Applies to DAST by detecting changes in fine motor performance over time, including pre- and post-treatment improvements or deterioration. | Determines whether the ML/DL model can detect meaningful changes over time, such as improvement or decline after an intervention. |
