# Supplementary material for: Clinical Applications and Measurement Properties of the Digitized Archimedes Spiral Drawing Test: A Scoping Review
Source: Mov Disord Clin Pract. 2025 Aug 7;12(11):1742–55. doi: 10.1002/mdc3.70278 (PMC12625189; doi:10.1002/mdc3.70278)
Supplement: Supplementary file 8 — Table S8. Studies that measure reliability. [file MDC3-12-1742-s002.docx]

## Table S8. Studies that Measure Reliability

| **First Author (Year)** | **Specific Population** | **Inter- or Intra- rater reliability** | **Test-retest reliability** | **Measurement error** | **Key Results** | **Clinical Utility** |
| --- | --- | --- | --- | --- | --- | --- |
| Aghanavesi S et al., (2017)^77^ | PD |  | X |  | Temporal Irregularity Score (TIS) Intraclass Correlation Coefficient (ICC) for three trials = 0.81 | TIS reliably differentiates advanced PD patients from nonpatients, with good test-retest reliability. |
| Aghanavesi S et al., (2017)^78^ | PD |  | X |  | Using scores derived by the SVM model, the ICC between the first two baseline measurements scores: Dyskinesia: 0.84. Treatment Response Scale (TRS): 0.51. UPDRS items: Lower ICCs, e.g., 0.17 for finger tapping (UPDRS #23) | Smartphone-based tapping and spiral drawing tests showed moderate validity and reliability in quantifying PD motor symptoms. While less reliable than clinical ratings, smartphone metrics offer a low-cost, scalable solution for remote monitoring and frequent symptom assessments. |
| Altmann VC et al., (2022)^56^ | Paralympic athletes |  | X |  | Bland-Altman analysis with limits of agreement (LOA) generally within two SD of the group mean | The Spiral Test exhibited sufficient test-retest reliability for most testing conditions. |
| Banaszkiewicz K et al., (2009)^11^ | PD |  | X |  | Test-retest reliability of the spiral drawing time was high (r=0.95 p<0.001) | Time of spiral drawing has high test-retest reliability, and it also correlates with other measures such as NHPT and BRAIN test, and UPDRS III (motor part) |
| Elble RJ et al., (2017)^93^ | ET |  | X | X | ICC: Tablet: 0.97 (95% CI: 0.91–0.99). FTM: 0.90 (95% CI: 0.76–0.96).  Minimum Detectable Change (MDC): Tablet: 51% of baseline tremor amplitude. FTM: 67% of baseline tremor amplitude. | The digitizing tablet reliably measures tremor severity in ET patients, with excellent test-retest reliability and sensitivity to small changes, aiding in monitoring progression and treatment efficacy. |
| Feys P et al., (2007)^12^ | MS |  | X |  | Spearman correlation coefficients: Circle Spiral: Radial velocity (SD-R): 0.76–0.90. Tangential velocity (SD-T): 0.74–0.95. Overall velocity (SD-DV): 0.75–0.90. Square Spiral: Horizontal velocity (SD-X): 0.48–0.83. Vertical velocity (SD-Y): 0.72–0.94. Overall velocity (SD-DV): 0.67–0.91. | The velocity-based metrics from circular and square spiral drawings reliably discriminate between MS patients with tremor, MS patients without tremor, and nonpatients. Digitized spirography offers clinical utility by reliably quantifying intention tremor severity in MS patients and distinguishing between MS patients with tremor, without tremor, and nonpatients, supporting diagnosis and monitoring. |
| Haubenberger D et al., (2011)^94^ | ET |  | X |  | Compared with the variation of the visual ratings, each computerized method showed a significantly lower variation across iteration (t test; p < 0.0001), whereas there was no difference between the time and space methods (P = 0.9). Coefficient of Variation (CV): For time and space methods, the CV was only 1.0% ± 0.9% (time) and 1.0% ± 1.1% (space). High correlations between digital scores and visual ratings across multiple time points during the ethanol intervention (r ≥ 0.8). | Compared to visual ratings, the digital methods demonstrated much greater consistency. Using metrics like velocity tremor peak amplitude and spectral peak frequency, the study demonstrated high reliability in capturing tremor characteristics. |
| Holcomb JM et al., (2023)^19^ | ET | X |  |  | Cohen’s Weighted Kappa values: ELANN vs. Neurologist 1 (rerated spirals): 0.93 (0.89–0.96). ELANN vs. Neurologist 2: 0.95 (0.92–0.97). Neurologist 1 rerated vs. Neurologist 2: 0.94 (0.90–0.97). | The CNN model can provide consistent, objective, and automated tremor evaluations. It has potential to standardize tremor severity scoring, reduce reliance on subjective expert ratings, and offer a scalable solution for use in both clinical and research settings, supporting ET management and monitoring. |
| Legrand AP et al, (2017)^32^ | ET | X | X | X | Test-retest ICC (2,1): Velocity (V) method:0.97. EMD: 0.96. URS: 1.00. URD: 0.99. Agreement between visual and computerized ratings ICC (2,1): V: 0.74. EMD: 0.74. URS: 0.84. URD: 0.85. Standard Error Estimates: Velocity (V): 0.38. EMD: 0.38. URS: 0.11. URD: 0.16. | Computerized methods provide a reliable, reproducible, and objective alternative to traditional visual ratings for assessing tremor severity. With strong agreement to expert ratings and minimal measurement error, these methods are valuable for accurately capturing tremor amplitude and frequency in clinical and research settings. |
| Lin PC et al, (2018)^95^ | PD & ET |  | X |  | ICC values for computerized metrics ranged between 0.88 and 0.97, indicating excellent test-retest reliability. | The computerized system produces consistent outputs across repeated tests under similar conditions and reliably measures tremor severity. |
| Longardner K et al, (2024)^30^ | ET |  | X |  | Test-retest reliability for both maximum and mean tremor amplitudes: Pearson correlation coefficients: Maximum amplitude: r [95% CI] = 0.80 [0.76, 0.83], p < 0.001, Mean amplitude: r [95% CI] = 0.91 [0.85, 0.95], p < 0.001 | The algorithm provides a valid, reliable, and sensitive tool for assessing kinetic tremor severity in ET. Digital measures reduce rater variability and offer objective continuous metrics, improving utility for clinical trials. Automated tremor assessments could facilitate remote monitoring and standardization in clinical settings, supporting treatment evaluations and therapeutic trials. |
| Memedi M et al, (2015)^62^ | PD | X | X |  | The Weighted Kappa coefficient between the ratings of the four raters and the Multilayer Perceptron (MLP)classifier was 0.65; slightly higher than the maximum coefficient found among the four raters.  Test-Retest Reliability of PCs across three trials: PC1: 0.75, PC2: 0.6, PC3: 0.64, PC4: 0.39. | MLP can reasonably well replicate visual interpretations of spirals by movement disorder specialists. MLP can separation between voluntary movements exhibited by HE subjects, and slow/involuntary movements found in PD patients and detection of differences in variabilities during spiral drawing between HE subjects and patients. Test-retest reliability was moderate to high for the primary PCs, indicating stable performance over repeated measurements. |
| Schallert W et al., (2022)^75^ | Patients with movement disorders |  | X | X | Relative Reliability (ICCs): Accuracy: 0.76–0.88; Speed: 0.20–0.86; Path Length: p<0.4; Absolute Reliability: Minimal Detectable Difference (MDD): Accuracy: 14%–38 across tasks. Speed and path length showed higher variability. | Tablet-based assessments provide an objective, reliable, and user-friendly method for evaluating upper limb motor function in patients with neurological disorders. These tools can facilitate remote monitoring, enabling patients to self-assess and share results with clinicians, promoting earlier detection of deterioration and better management of neurological conditions. |
| Schuhmayer N et al., (2017)^96^ | ET |  | X |  | Tremor frequencies across repeated spiral tasks: ICC = 0.959 (dominant hand), 0.971 (non-dominant hand). | Digitizing tablet-based assessments provide a reliable, valid, and non-invasive method for quantifying ET in kinetic and postural tasks. These methods can improve tremor tracking over time, offering valuable insights into the functional impact of treatments and supporting personalized care strategies for patients with ET. |
| Westin J et al., (2010)^64^ | PD | X | X |  | Strong interrater reliability (*r*=0.87) and excellent agreement between WSTS and manual ratings (*r*=0.91). Test-Retest Reliability: WSTS: *r*= 0.77 SDDV: *r*=0.79 | The WSTS provides an objective, reliable, and robust tool for assessing motor impairments in PD, suitable for use in clinical trials and telemedicine settings. The method is particularly effective for capturing motor fluctuations and dyskinesias, making it valuable for monitoring treatment effects, especially in advanced PD. |
