# Supplementary material for: Clinical Applications and Measurement Properties of the Digitized Archimedes Spiral Drawing Test: A Scoping Review
Source: Mov Disord Clin Pract. 2025 Aug 7;12(11):1742–55. doi: 10.1002/mdc3.70278 (PMC12625189; doi:10.1002/mdc3.70278)
Supplement: Supplementary file 9 — Table S9. Studies that measure validity. [file MDC3-12-1742-s010.docx]

## Table S9. Studies that Measure Validity

| **First Author (Year)** | **Construct validity (Convergent validity)** | **Construct validity (Discriminant or Known-group validity)** | **Criterion validity (Concurrent validity)** | **Key Results** | **Clinical Utility** |
| --- | --- | --- | --- | --- | --- |
| Aghanavesi S et al., (2017)^77^ | X | X |  | TIS successfully **differentiated advanced PD patients from HC** (p=0.02) but **did not distinguish early-stage** patients or the entire PD group from controls (p=0.07). Correlations between TIS and clinical ratings (UPDRS items and Dyskinesia Scale) were weak (r=-0.10 to −0.31). This shows **limited convergent validity.** | TIS proved to be a reliable measure, but its utility for assessing short-term treatment effects or aligning with clinical ratings was limited. |
| Aghanavesi S et al., (2017)^78^ | X | X |  | The study correlates smartphone-derived scores with UPDRS (r=0.47–0.64), demonstrating **moderate convergent validity**. Construct validity is supported by **significant group differences** between PD patients and nonpatients (p<0.005). | While less reliable than clinical ratings, smartphone metrics offer a low-cost, scalable solution for remote monitoring and frequent symptom assessments. This approach could complement clinician assessments, especially in resource-limited settings or for longitudinal symptom tracking |
| Almeida MF et al., (2012)^28^ | X | X |  | The study shows a **strong linear correlation between LDA-values and age** (R²=0.81), validating the sensitivity of the feature to physiological tremor changes. | The study demonstrates the LDA-value's correlation with age, suggesting potential clinical applications for distinguishing physiological from pathological tremor. |
| Almeida MF et al., (2010)^36^ | X | X |  | LDA-value showed to be **linearly correlated with age** for the group of clinically healthy subjects (r= 0.83). The results found in our study suggest a degradation of the motor control with age under a specific task. | The LDA-value's correlation with age suggests potential clinical applications, though it does not directly validate DAST for diagnostic purposes. |
| Baek H et al., (2024)^58^ |  |  | X | Validated accelerometer-based measurements of tremor before, during, and after the MRgFUS procedure. This was demonstrated through moderate to **strong correlations between the quantitative drawing scores and accelerometer metrics** in both ET and PD patients: ET group: R² = 0.51, p = 0.029; PD group: R² = 0.85, p = 0.0008 | The study demonstrates clinical utility by showing how these drawing metrics correlate with treatment effects and can be used for remote, long-term follow-up. The findings indicate the potential of the DAST to monitor treatment durability in clinical settings. |
| Banaszkiewicz K et al., (2009)^11^ | X | X |  | Spiral Drawing time was **significantly correlate with motor measures**. UDPRS III: r=0.64, p=0.0007, BRAIN: r=-0.52, p=0.001, 9HPT: r=0.55, p=0.03. There is also a significant difference between PD and healthy controls (p<0.001) | Computerized measure (drawing time) shows significant correlation with other objective measures. This is a simple and inexpensive method for Bradykinesia and can be used in clinical evaluation and pathophysiology and epidemiological studies |
| Bui HT et al., (2017)^43^ |  | X |  | Frequency analysis revealed reduced movement smoothness in ARSACS patients, with specific bands (1.2–1.7 Hz) **effectively distinguishing them from HC.** Mean error achieved 95.9% classification accuracy, which frequency analysis improved to nearly 100%. | The frequency analysis and measurement of incoordination offer indirect evidence of clinical utility, supporting the DAST’s potential to track coordination changes in neurodegenerative conditions. |
| Chen KH et al., (2018)^103^ |  | X |  | This study confirms the validity of **visuomotor feedback velocity metrics in distinguishing PD from ET patients**. PD patients showed slower absolute velocities during static tracing (T1, p < 0.05) but matched guiding velocities in dynamic tracing (T2). ET patients exhibited higher velocities in T2 (p < 0.05) but struggled to match the guiding point. No significant correlation was found between tremor severity (FTM for ET), motor symptoms (UPDRS Part III for PD), and tracking performance (R < 0.5, p > 0.05), indicating velocity control reflects distinct motor functions. | The study demonstrates clinical utility by showing that differences in velocity control under visual guidance can help differentiate PD from ET, potentially aiding in diagnostic applications. |
| Creagh AP et al., (2020)^39^ | X | X |  | The study found **significant correlations between DaS Test metrics (e.g., drawing velocity, error, entropy) and 9HPT times** (R² = 0.39 for the dominant hand, R² = 0.41 for the non-dominant hand), with notable differences in drawing features between HC and PwMS (abnormal U function). | The results show that the smartphone based DaS Test can predict 9HPT times with reasonable accuracy, suggesting potential clinical utility for remote monitoring of upper extremity function in MS patients. |
| Danna J et al., (2019)^68^ |  | X | X | Key kinematic variables, including **velocity peaks and pen altitude variations, differed significantly between groups** (PD vs. HC, and PD "ON" vs. "OFF" medication), reflecting motor impairments and medication effects. A significant correlation (𝜌 = 0.57, p = 0.01) between medication-induced changes in velocity peaks and UPDRS scores supports criterion validity. | The study shows potential clinical utility by identifying kinematic changes in motor function related to PD that could be used for monitoring disease progression and medication effects. |
| DelMastro HM et al., (2018)^41^ | X |  |  | The study found SEGRT (Segment Rate) analysis of digital spirals **more sensitive (84.6%) in detecting tremor in MS** patients than manual Archimedes Spirals (61.5%). SEGRT and other digital measures correlated significantly w**ith clinical tests (9HPT, Box-and-Block Test)** | The study investigates the clinical utility of SEGRT for assessing tremor severity in MS, showing that digital measures offer more sensitivity and could be used in routine clinical assessments. This supports the clinical utility of the measurement. |
| Elble RJ et al., (1996)^7^ |  |  | X | The digitizing tablet produced reproducible tremor amplitude and frequency measurements with intertrial **correlations comparable to accelerometry** (r=0.85−0.91). | The study validates the digitizing tablet as an objective, cost-effective tool for quantifying tremor severity in ET patients, with measurements correlating significantly with the TRS. This non-invasive method is practical for monitoring tremor progression and treatment effects. |
| Elble RJ et al., (2017)^93^ |  |  | X | The tablet's tremor measurements demonstrate a **strong logarithmic relationship** (r = 0.94) with FTM tremor ratings, demonstrating criterion validity. | The study confirms the digitizing tablet's clinical utility in quantifying tremor severity in ET patients, with strong correlations to FTM ratings. Its objective, precise assessments offer a cost-effective complement to traditional ratings, improving tremor monitoring in clinical and research settings. |
| Feys P et al., (2007)^12^ | X | X |  | Spiral features **strongly correlated with clinical tremor severity (FTRS) and functional assessments (TEMPA, 9HPT**), with strong relationships: SD-Y and FTRS total (r = 0.84). These features effectively distinguished MS tremor patients from those without tremor and HC (p < 0.0001). | The study validates digitized spiral as an objective, reliable tool for assessing intention tremor severity in MS, aiding in monitoring disease progression and treatment efficacy in clinical settings. |
| Fujiwara K et al., (2023)^13^ |  | X |  | The **dual-task method showed significant differences** in drawing performance between the MCI and HC, with MCI participants showing greater deviation from the centerline and higher inward displacement during the drawing task. The system demonstrated high effectiveness, achieving 90% sensitivity and 75% specificity in detecting MCI. | Dual task testing of the spiral drawing and counting tasks offers a complementary assessment alongside traditional diagnostic tools, facilitating early intervention and improving cognitive care. |
| Galli M et al., (2014)^98^ |  | X |  | PD patients (off medication) exhibited significantly **smaller spiral dimensions and slower drawing speeds** compared to nonpatients (p < 0.05). Significant improvements in these metrics were observed in the PD "on" condition compared to the "off" condition (p < 0.05). | The study shows that spiral dimension and velocity profiles improve with levodopa treatment, highlighting the DAST's responsiveness and its potential role in monitoring treatment efficacy. |
| Haubenberger D et al., (2011)^94^ |  | X | X | Digital spiral scores showed **strong correlations with visual spiral ratings** (r = 0.866 for time, r = 0.870 for space, p < 0.0001) and clinical FTM scores (time: r = 0.922, p < 0.0005; space: r = 0.829, p < 0.05). They reliably **captured tremor severity during the ethanol challenge**, aligning with visual ratings and FTM scores. All methods detected significant tremor reduction within 15 minutes (p < 0.05), peaking at 45 minutes (p < 0.01). | Compared to visual assessments, digital analysis proved more consistent and sensitive to subtle treatment effects, highlighting its utility as a precise outcome measure for clinical trials and patient monitoring in ET. |
| Hermle D et al., (2024) | X | X |  | **Strong correlations with established clinical outcomes: SARA** (\|ρ\| = 0.60–0.81 for various tasks), **FARS-ADL** (\|ρ\| = 0.64), particularly for upper limb-specific tasks, 9HPT (\|ρ\| = 0.63–0.81). All tasks significantly **discriminated ataxia patients from HC**, with AUC values exceeding 0.7. | The study highlights the DAST’s utility in assessing motor deficits and tracking ataxia severity, making it a valuable tool for both clinical assessment and research applications in degenerative cerebellar diseases |
| Hess CW et al., (2014) |  | X |  | Spiral drawing **variability significantly differentiated ET, PD, and HC groups** (p < 0.001) and correlated with UPDRS scores in PD. **ET patients had the largest deviations**, reflecting tremor amplitude and irregularity, while **PD patients showed irregular speed and inconsistent acceleration**, indicating bradykinesia and tremor. | Digital spiral analysis shows promise as an objective tool for diagnosing functional tremor, offering clinicians a non-invasive method to distinguish psychogenic from organic tremors through quantifiable measures. |
| Hoogendam YY et al., (2015)^55^ |  | X |  | The study found significant motor impairments in chemotherapy-exposed breast cancer survivors (20 years post-treatment) compared to women without cancer: **slower movement times** (p = 0.027), **longer spirals** (p = 0.010), **greater speed variability** (p = 0.049), and **more return movements** (p = 0.047). These effects worsened with age, suggesting a compounding impact of aging on fine motor function. | DAST is a sensitive, non-invasive tool for detecting fine motor impairments in cancer survivors, helping clinicians monitor motor health and enable early interventions to support daily function and quality of life. |
| Hoogendam YY et al., (2014)^57^ | X | X |  | **Older age was linked to poorer performance on all spiral-drawing measures** (p < 0.001), with participants over 75 showing greater deviations from the template, reflecting a quadratic relationship with age (p < 0.01). Larger gray matter volume was associated with less speed variability (β = -0.13, p < 0.05), fewer template deviations (β = -0.20, p < 0.05), and fewer template crossings (β = -0.12, p < 0.05). Larger white matter volume correlated with reduced speed variability (β = -0.15, p < 0.05) and fewer deviations (β = -0.14, p < 0.05). | DAST in this context lies in its potential as a non-invasive, quantitative tool to monitor fine motor changes due to aging, providing valuable insights for early detection of motor impairment in older adults and facilitating interventions that may slow motor decline. |
| Hsu AW et al., (2009)^14^ |  | X |  | NPC patients exhibited **slower speeds, less acceleration, greater loop width variability, and increased tremor amplitude** (all p < 0.001), indicating substantial impairments in motor control. | The study shows that DAST metrics, especially loop width variability and tremor characteristics, reliably indicate motor impairments in NPC, highlighting its utility for tracking motor dysfunction in neurodegenerative conditions. |
| Ishii N et al., (2020)^48^ |  | X |  | Spiral metrics differed significantly among HC, ET, and CD groups for **spiral length and deviation area** (p < 0.001), with the CD group showing the largest deviations, reflecting more severe motor dysfunction. | A smartphone app offers a non-invasive, objective tool for assessing tremor severity, aiding diagnosis, and improving neurological assessments, especially in settings with limited equipment. It can guide referrals and monitor tremor progression over time. |
| Koppelmans V et al., (2024)^53^ | X | X |  | **Spiral tracing metrics** (e.g., movement time, speed variability, template deviation) **showed no significant differences between HC, MCI, and AD groups**. While TMT-A completion time and grip strength correlated with amyloid-β deposition, **spiral drawing metrics did not correlate with AD biomarkers.** | Spiral drawing performance is less sensitive to the underlying pathophysiological changes associated with AD compared to other motor tasks or cognitive assessments. It highlights a limitation of the DAST in reflecting the neurobiological changes associated with cognitive decline in the MCI and AD populations examined in this study. |
| Kraus PH et al., (2010)^101^ |  |  | X | Regression analysis show spiralometry-derived **tremor amplitude measurements are highly significant associated with the Bain and Findley rating**s (r² = 88.9%, p < 0.0001) | Spiralometry technique is inexpensive and objective. Data from spiralometry has higher resolution and can lead to lower sample size required for clinical trial. |
| Legrand AP et al., (2017)^32^ | X |  |  | The computerized methods **(e.g., velocity-based analysis, unravelling methods, and empirical mode decomposition) are highly correlated with the Bain** tremor rating scale with correlation coefficients (r=0.79–0.87). | New numerical methods outperform velocity analysis, are more reproducible than expert ratings, and offer an easy-to-use alternative system. They also provide complementary data on tremor amplitude and frequency, enhancing diagnostic accuracy. |
| Lin PC et al., (2018)^95^ |  |  | X | Significant correlations were found between the digital parameters and VRS scores across test paradigms: S1 (traced along a given spiral): Correlations ranged from R=0.872 to 0.973 (all p < 0.01); S3 (traced along a guiding point): Correlations ranged from R=0.944 to 0.967 (all p < 0.01); **Lower correlations were observed in S2 (freehand drawing)** | The study demonstrated the feasibility of using digital graphic tablets with different guiding paradigms can be used to quantify the temporal and spatial characteristics of tremors automatically. The spiral drawing indices can be used for easier and real-time tremor severity evaluation in clinical environments. |
| Liu X et al., (2005)^66^ | X |  |  | **Significant correlations demonstrate alignment between the SD-DV measur**e and established clinical assessments of dyskinesias and motor severity. Bain Dyskinesia Scale: r=0.65, p<0.01; UPDRS medication-on and medication-off scores r=0.63 and 0.64, respectively. | The study's method quantified arm dyskinesias during drawing tasks, with potential to assess other involuntary movements like intention tremor or chorea, aiding diagnosis and treatment evaluation. |
| Longardner K et al., (2024)^30^ |  |  | X | **Automated tremor amplitude measures (mean and maximum) strongly correlated with manual measurements** (r = 0.91 [0.85, 0.95], p < 0.001). Both mean and max amplitudes from the algorithm also significantly associated with TETRAS score (p<0.0006) when TETRAS score was greater than 1.33, but no association when TETRAS were less than 1.33 (p=0.05) | The digital measure provides a valid, clinically relevant, easy-to administer outcome measure for maximum and mean tremor amplitude that can be useful for both clinical care and future clinical trials in ET. |
| Louis ED et al., (2012)^80^ | X | X |  | **The SWVI, a kinematic measure from digital spiral analysis, correlated with total tremor score** (r = 0.41, p < 0.001), arm intention tremor (r = 0.27, p = 0.001), and tandem gait missteps (r = 0.16, p = 0.06). **ET cases had higher SWVI scores than controls** (median 0.46 vs. 0.30, p < 0.001), with higher scores linked to head intention tremor (p < 0.001). | The study provides strong evidence for the SWVI’s utility as a digital marker of cerebellar dysfunction. |
| MacWilliams et al. (2021) |  | X |  | **Statistically significant difference in RMS and tremor amplitude scores between cases and controls** | Collecting and analyzing spiral data using a simple, scalable digital approach that detects fine motor deficits and tremors at rates comparable to traditional methods offers significant clinical utility for large-scale and repeated volunteer assessments before and after candidate treatments. |
| Memedi M et al., (2015)^62^ | X | X |  | Groups differed significantly in all PCs except PC3. Convergent validity showed **moderate to strong correlations (r = 0.56–0.69) with specialist ratings**, and construct validity was supported by **significant PD vs. control differences** (p < 0.05). | The method effectively distinguishes PD-specific and treatment-induced motor symptoms, aligning with specialist visual assessments. It offers objective summary scores to aid treatment decisions, optimizing functional on time while minimizing Off episodes and dyskinesia. |
| Merchant SH et al., (2018)^81^ |  | X |  | Significant differences in **SWVI (p = 0.0021) and DoS (p = 0.0107**) were observed **between patients with early DBS tolerance and satisfactory outcomes**. Using a 0.40 SWVI cutoff, sensitivity was 100% and specificity 89% for predicting early DBS tolerance. | The SWVI proved to be a strong predictor of early DBS tolerance, offering objective, reproducible data on pre-operative tremor and cerebellar dysfunction, supporting its use in predicting outcomes and tailoring treatments. |
| Rajan R et al., (2021)^104^ | X | X | X | Algorithm-derived variables, MD and SD-TV, **correlated with Bain and Findley spiral score**s (ρ = 0.491, p < 0.001 for MD), **FTM-TRS Part B** (ρ = 0.260, p = 0.032 for MD), and **accelerometric measures of postural tremor** (ρ = 0.366–0.402, p < 0.001). MD was higher in the tremor group (48.9 ± 26.3) than HC (26.4 ± 5.3, p < 0.001). Loop width variables significantly distinguished PD spirals from ET, DT, and HC (p < 0.001). | An automated algorithm for analyzing hand-drawn spirals provides clinically meaningful, robust metrics with minimal resources. It is useful in resource-limited settings, neurology clinics, and large-scale surveys for detecting and quantifying tremor. |
| Ratliff J et al., (2018)^46^ | X | X |  | Spiral analysis indices, including **ResThetaR (r = 0.55, p = 0.005) and 2nd Order Smoothness (r = 0.53, p = 0.007), correlated with BFM-Motor scores and significantly differed between dystonia patients and controls** (p < 0.001). | Using spiral analysis may enhance our understanding and help identify other genetically identifiable forms of dystonia. |
| Roth N et al., (2021)^102^ |  | X |  | Significant medium-to-high correlations (p < 0.01) were found between spiral and vertical line deviations. **ET patients showed greater spiral radius deviations than HC (p < 0.05).** | Different drawing directions affect performance, with vertical flexion-extension movements dominating spiral and line tasks. This insight could help optimize drawing direction and hand posture for individual performance. |
| San Luciano M et al., (2016)^99^ | X | X |  | **DoS, mSp, SWVI, and Smoothness** significantly differentiate between PD and control groups. PD and control groups (p<0.001 for multiple indices), Early PD (≤5 years) and control groups (p<0.05). A model using all indices had high discriminative validity (sensitivity = 0.86, specificity = 0.81). **Discriminative validity was maintained in patients with early PD**. Spiral indices did not vary according to motor subtype except for SWVI, which was significantly higher in the intermediate or postural instability-gait disorder (PIGD) group when compared to tremor-dominant (TD) group (TD vs. PIGD p = 0.007). | The study highlights the ease of use, cost-effectiveness, and non-invasive nature of digitized spiral analysis, as well as its ability to assess proximal and distal arm movements without restricting natural motion. |
| Saunders-Pullman R et al., (2008)^100^ | X |  |  | Spiral indices, including **2nd OrderSm, DoS, mSp, correlated with total UPDRS** scores and domain sub-scores (r = 0.29–0.42, p < 0.01). Specific indices linked to motor symptoms were 2nd OrderSm (bradykinesia, rigidity, action tremor, not rest tremor), mSp (rigidity, bradykinesia, action tremor), and DoS (rest tremor effects). | Spiral analysis offers objective, continuous metrics for subtle motor impairments, complementing the UPDRS scale and detecting small changes in early PD where UPDRS may be less sensitive. |
| Schallert W et al., (2022)^75^ |  | X |  | The study found significant performance differences between HC and neurological patients. **Accuracy showed excellent discriminative validity** (AUC > 0.93) across tasks, while **speed and path length had mixed reliability and weaker validity in some tasks.** | Tablet-based tests may outperform clinical scales in responsiveness, motivate patients with performance feedback, and enable easy sharing of results with physicians for frequent monitoring and early detection of deterioration. |
| Schuhmayer N et al., (2017)^96^ | X |  | X | Spiral drawing **tremor severity strongly correlated with TETRAS scores** (ρ = 0.85, p < 0.001). Spiral tremor frequencies were **>2 Hz higher than postural tremor** frequencies (p < 0.001). Tremor amplitude declined during tasks, likely due to learning and adaptation. The overall agreement of tremor frequencies between digitizing based methods was high (spiral vs. e-lines ICC=0.783, spiral vs. dot-approximation ICC=0.711, e-lines vs; dot-approximation ICC=0.660). | Variability in tremor frequency and amplitude may offer insights into the neurophysiological mechanisms of action tremors, while isolated measures have limited diagnostic value. |
| Starita S et al., (2022)^31^ |  | X |  | **Spectral features** (e.g., frequency peaks, bandwidth, and power distribution) **are better at differentiating PD patients from HC than kinematic features**. PD patients show a clear frequency peak (~6.5 Hz) in tremor, while controls show a broader, less distinct frequency range. | Features extracted from spectral analysis offer a valid inquiring approach to characterize parkinsonian motor behavior, thus support it as a tool for differential diagnosis of tremor-presenting patients. |
| Toffoli S et al., (2023)^92^ | X | X |  | The pen-based system distinguished PD patients from HC through differences in **smoothness, frequency, and force**. Tremor indicators (e.g., 4–7 Hz angular velocity power) correlated with **UPDRS III resting tremor scores**, while fluency indicators correlated with overall disease severity (ρ ≈ 0.4–0.5). | The pen-based system identified 25 indicators distinguishing PD patients from controls, moderately correlating with clinical scales (UPDRS, H&Y) and detecting subtle motor impairments missed by clinical scores. As a practical, sensitive tool, the smart pen can complement evaluations, support early detection, monitor disease progression, and integrate with telemedicine for personalized care. |
| Ueda N et al., (2014)^45^ | X | X |  | **GA significantly correlated with total SARA scores** (r = 0.660, p < 0.001), sub-scores, and cerebellar volume (r = −0.577, p < 0.001), supporting its validity in quantifying ataxia and structural correlates. | The GA measure from spiral drawing is a simple, objective tool for quantifying upper-limb ataxia in spinocerebellar degeneration. Strongly correlated with SARA and cerebellar volume, it offers a cost-effective method for tracking disease progression and treatment efficacy, suitable for large-scale studies and routine clinical use. |
| Ulmanová O et al., (2007)^105^ |  | X | X | Tremor magnitude **significantly differentiated ET patients from HC** across tasks (e.g., spiral drawing, p<0.01). The mean tremor magnitude for all tasks also **differed significantly between the groups** (p<0.0001). Tremor magnitude **correlated strongly with total FT scale** scores and specific visual assessments (p<0.01), aligning with clinical observations of tremor severity. | The tremor magnitude index is a simple, objective measure that distinguishes ET patients from controls, correlates with clinical ratings, and supports assessing daily activity disability in clinical and research settings. |
| Westin J et al., (2010)^64^ | X | X | X | WSTS **showed high agreement with manual ratings** (r = 0.89, ±1.5 units) and **moderate correlations with UPDRS total** (r = 0.41) and subsection II (r = 0.51), indicating alignment with gold standards but limited scope for broader motor impairments. | The study highlights WSTS as a reliable tool for assessing motor impairments in PD, especially for telemedicine and remote monitoring, but its reliance on patient adherence and inability to differentiate specific symptoms limit its broader use. |
