# Supplementary material for: Clinical Applications and Measurement Properties of the Digitized Archimedes Spiral Drawing Test: A Scoping Review
Source: Mov Disord Clin Pract. 2025 Aug 7;12(11):1742–55. doi: 10.1002/mdc3.70278 (PMC12625189; doi:10.1002/mdc3.70278)
Supplement: Supplementary file 10 — Table S10. Studies that measure responsiveness. [file MDC3-12-1742-s003.docx]

## Table S10. Studies that Measure Responsiveness

| **First Author (Year)** | **Study Aims** | **Key Results** | **Clinical Utility** |
| --- | --- | --- | --- |
| Aghanavesi S et al., (2017)^77^ | To evaluate the sensitivity of TIS to a single dose of levodopa over a course of test trials. | From 15 to 60 minutes post-dose, the effect sizes increase consistently (close to 0.08). Between 100 and 300 minutes, the effect sizes decrease, showing the wearing-off effect of levodopa. At 80 minutes, the effect size is unexpectedly smaller than expected (0.03), indicating potential variability in treatment response or measurement. | While TIS has limited sensitivity to single-dose levodopa, it may be more suitable for assessing long-term treatment effects, especially for advanced therapies like LCIG pumps. TIS may serve as a complementary digital biomarker alongside traditional clinical assessments to provide objective, high-frequency insights into motor function |
| Aghanavesi S et al., (2017)^78^ | To evaluate the ability of both clinical and smartphone-based measures to detect treatment-related changes in motor symptoms in response to levodopa administration. | PC1 captured trends in motor symptom improvements and deteriorations across the levodopa test cycle, including transitions between "Off," "On," and "On with dyskinesia" states | While less reliable than clinical ratings, smartphone metrics offer a low-cost, scalable solution for remote monitoring and frequent symptom assessments. This approach could complement clinician assessments, especially in resource-limited settings or for longitudinal symptom tracking |
| Baek H et al., (2024)^58^ | To evaluate the ability of quantified drawing analysis to detect treatment-related improvements in tremor following MR-guided focused ultrasound (MRgFUS) thalamotomy. | Significant decreases in composite scores for spiral and line drawings were observed immediately after MRgFUS in both ET and PD patients. ET: 𝑝=0.0002, PD: p=0.12 (not statistically significant) | Quantitative drawing analysis demonstrated strong responsiveness to MRgFUS treatment, making it a valuable tool for objectively monitoring tremor improvements. Its ability to capture long-term treatment durability through remotely administered tests (e.g., mailed drawing sheets) offers a cost-effective and scalable solution for follow-up care in patients with ET and PD. |
| Galli M et al., (2014)^98^ | To evaluate the ability of quantitative spiral analysis to detect changes in motor performance before and after levodopa treatment in Parkinson’s disease (PD) patients. | Dimension of the Spiral (D): p<0.05 for differences between OFF and ON conditions. Angular coefficients of velocity profile regression were higher in the ON condition than OFF, indicating improved motor consistency. | By measuring metrics such as spiral dimension and velocity profiles, the DAST provides a non-invasive, quantifiable method for assessing motor symptoms like micrographia and bradykinesia. The study shows that these metrics improve with levodopa treatment, highlighting the DAST's responsiveness and its potential role in monitoring treatment efficacy. Additionally, the DAST's ability to distinguish between PD patients and nonpatients supports its use in clinical assessments of motor impairments, making it a valuable tool for both diagnosis and ongoing patient management. |
| Haubenberger D et al., (2011)^94^ | To evaluate the ability of digital spiral analysis to detect changes in tremor severity after ethanol administration in patients with ET | Tremor intensity was significantly reduced after ethanol administration, with the maximum reduction occurring at 45 minutes post-administration. Computerized spiral scores captured tremor reduction effectively, demonstrating a mean reduction of tremor by approximately 50% at 15 minutes post-ethanol, which was consistent across methods (time and space). | Compared to visual assessments, digital analysis proved more consistent and sensitive to subtle treatment effects, highlighting its utility as a precise outcome measure for clinical trials and patient monitoring in ET. |
| Radmard S et al., (2021)^106^ | To evaluate the ability of computerized spiral analysis to detect changes in motor function after deep brain stimulation (DBS) targeting the subthalamic nucleus (STN) or ventral intermediate thalamus (Vim) in Parkinson’s disease (PD) patients. | Severity: Improved significantly after STN DBS (p<0.001) and Vim DBS (p<0.007). Smoothness: Improved significantly after both STN (p<0.001) and Vim DBS (p=0.005). Tremor: Improved after STN DBS (p<0.001) and Vim DBS (p=0.01), with a greater effect observed after Vim DBS. Variability: Improved only with Vim DBS (p=0.01), not STN DBS (p=0.66). Tightness: No significant improvement with either STN or Vim DBS, consistent with findings that DBS does not ameliorate micrographia. | Spiral analysis can be utilized to objectively evaluate the effects of  DBS in PD subjects, providing a window on relevant clinical features  that change post-operatively. |
